# The Chromatin Remodeler BPTF Activates a Stemness Gene-Expression Program Essential for the Maintenance of Adult Hematopoietic Stem Cells

**DOI:** 10.1016/j.stemcr.2018.01.020

**Published:** 2018-02-15

**Authors:** Bowen Xu, Ling Cai, Jason M. Butler, Dongliang Chen, Xiongdong Lu, David F. Allison, Rui Lu, Shahin Rafii, Joel S. Parker, Deyou Zheng, Gang Greg Wang

**Affiliations:** 1 Lineberger Comprehensive Cancer Center, University of North Carolina at Chapel Hill School of Medicine, Chapel Hill, NC 27599, USA; 2 Department of Biochemistry and Biophysics, University of North Carolina at Chapel Hill School of Medicine, Chapel Hill, NC 27599, USA; 3 Department of Medicine and Ansary Stem Cell Institute, Weill Cornell Medical College, New York, NY 10065, USA; 4 Laboratory of Biochemistry and Molecular Biology, Rockefeller University, New York, NY 10065, USA; 5 Department of Neuroscience and Neurology, Albert Einstein College of Medicine, Bronx, NY 10461, USA

**Keywords:** Bptf, hematopoietic stem cells, chromatin remodeler, Meis1, Pbx1, Mn1, DNA accessibility, NURF, AP1 complex

## Abstract

Self-renewal and differentiation of adult stem cells are tightly regulated partly through configuration of chromatin structure by chromatin remodelers. Using knockout mice, we here demonstrate that bromodomain PHD finger transcription factor (BPTF), a component of the nucleosome remodeling factor (NURF) chromatin-remodeling complex, is essential for maintaining the population size of hematopoietic stem/progenitor cells (HSPCs), including long-term hematopoietic stem cells (HSCs). *Bptf-*deficient HSCs are defective in reconstituted hematopoiesis, and hematopoietic-specific knockout of *Bptf* caused profound defects including bone marrow failure and anemia. Genome-wide transcriptome profiling revealed that BPTF loss caused downregulation of HSC-specific gene-expression programs, which contain several master transcription factors (*Meis1*, *Pbx1*, *Mn1*, and *Lmo2*) required for HSC maintenance and self-renewal. Furthermore, we show that BPTF potentiates the chromatin accessibility of key HSC “stemness” genes. These results demonstrate an essential requirement of the chromatin remodeler BPTF and NURF for activation of “stemness” gene-expression programs and proper function of adult HSCs.

## Highlights

• The chromatin remodeler gene *Bptf* shows preferential expression in primitive HSPCs
• Bptf ensures maintenance and functionality of HSCs in a cell-autonomous manner
• Bptf is required for the activation of HSC “stemness” genes including master TFs
• Hematopoietic-specific loss of Bptf results in bone marrow failure and anemia

## Teaser

Wang and colleagues show that a chromatin remodeler, BPTF, sustains appropriate functions of hematopoietic stem/progenitor cells (HSPCs). BPTF loss causes bone marrow failure and anemia. The authors further define a BPTF-dependent gene-expression program in HSPCs, which contains key HSC stemness factors. These results demonstrate an essential requirement of the BPTF-associated chromatin remodelers for HSC functionality and adult hematopoiesis.

## Introduction

Appropriate self-renewal and differentiation of adult stem cells are essential for tissue homeostasis and are tightly controlled by various cellular and molecular mechanisms, including the dynamic regulation of chromatin structure by ATP-dependent chromatin-remodeling complexes (Kadoch and Crabtree, 2015, Wang et al., 2007). These remodelers use energy produced from ATP hydrolysis to configure nucleosomal positioning and modulate DNA accessibility. Such a process ensures fidelity of crucial gene-expression programs during developmental processes such as lineage specification. In support of this pathway contributing to cell fate determination, somatic mutation of chromatin remodeler genes is common in human disease, including cancer (Kadoch and Crabtree, 2015, Wang et al., 2007).

Bromodomain PHD finger transcription factor (BPTF) is a core and largest component of the conserved, multi-subunit nucleosome remodeling factor (NURF) complex (Landry et al., 2008, Ruthenburg et al., 2011). NURF loosens condensed chromatin to promote DNA accessibility and target gene activation (Ruthenburg et al., 2011, Schwanbeck et al., 2004, Wysocka et al., 2006). While global knockout of *Bptf* in mice leads to lethality on embryonic day 8.5, demonstrating its requirement for early development (Landry et al., 2008), clinical studies reveal loss-of-function mutation of *BPTF* in individuals with syndromic neurodevelopmental anomalies (Stankiewicz et al., 2017). Furthermore, BPTF was recently shown to be critical for the maintenance or differentiation of mammary gland stem cells (Frey et al., 2017), melanocytes (Dar et al., 2016, Large et al., 2016), and T cells (Landry et al., 2011, Wu et al., 2016). BPTF contains two motifs in its C terminus, a PHD finger and a bromodomain that bind to histone H3 lysine 4 trimethylation (H3K4me3) and histone acetylation, respectively (Chi et al., 2010, Ruthenburg et al., 2011, Wysocka et al., 2006). Deposition of these two modifications occurs partly via the histone methyltransferase MLL/KMT2A and associated histone acetyltransferases (Dou et al., 2005). While previous works detail the essential role for KMT2A in regulation of hematopoietic and neuronal stem cells (Artinger et al., 2013, Jude et al., 2007, Lim et al., 2009), the specific contributions of BPTF remain undefined in this process.

Using knockout mice, we here show BPTF as a crucial chromatin regulator of hematopoietic stem cells (HSCs). Reconstitution assays demonstrate that *Bptf-*null HSCs exhibited the decreased repopulating capacity, causing severe hematopoietic defects. Our genomic profiling shows that ablation of BPTF in hematopoietic stem/progenitor cells (HSPCs) leads to decreased expression of an HSC-specific gene-expression program, which includes a master transcription factor (TF) regulatory node (*Meis1*, *Pbx1*, *Mn1*, and *Lmo2*) known to be crucial for HSC self-renewal and function. We also find that BPTF potentiates the chromatin accessibility of these HSC TF genes. Collectively, our results support a vital requirement of the BPTF chromatin remodeler for the maintenance of adult HSPCs and for the activation of a gene transcription program essential for HSC functions.

## Results

### Maintenance of Adult HSPCs, Including Long-Term HSCs, Requires *Bptf* Expression

**Figure 1:**
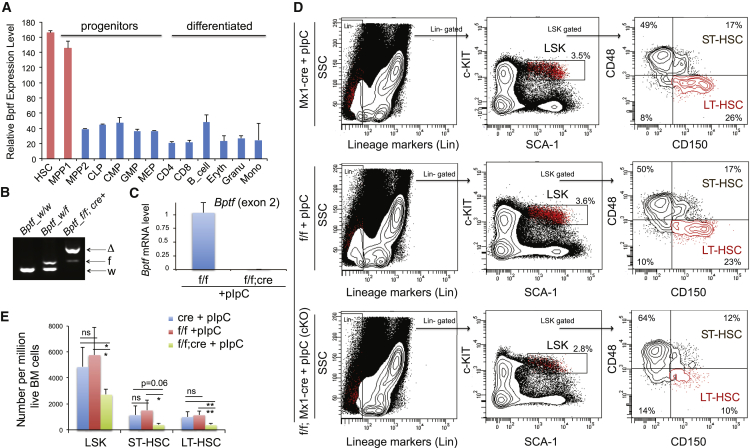
Maintenance of Adult HSPCs Including LT-HSC Requires BPTF (A) *Bptf* expression in hematopoiesis (see also Figures S1A and S1B). (B and C) Genotyping (B) and RT-PCR (C; n = 3 biological replicates) confirm deletion of the *Bptf* exon 2 in total bone marrow (BM) 1 week after cre induction. w, wild-type; f, floxed; Δ, deleted (*Bptf*^*cKO*^). (D and E) FACS (D) and summary (E) of percentages of the LSK and LT-HSC cells in the BM, 4 weeks after cre induction in the *Bptf*^*cKO*^ (f/f; cre, n = 5 mice) or control littermates with *Bptf*^*f/f*^ (f/f) or *Mx1*-cre (cre) alone (n = 4 mice). Numbers in (D) indicate the percentage of gated cells. Plots in (E) are mean ± SD, with statistical analysis defined by two-tailed Student's t test: ns, not significant; ^∗^p < 0.05; ^∗∗^p < 0.01.

Using transcriptome datasets of hematopoiesis (Bock et al., 2012, Seita et al., 2012), we found *Bptf* preferentially expressed in the primitive HSPC compartment (Figures 1A, S1A, and S1B). To study the role of BPTF in HSPCs, we produced inducible knockout mice (*Bptf*^*flf*^;*Mx1-*cre) designed to ablate *Bptf* from the bone marrow (BM) upon activation of *Mx1*-cre by polyinosinic-polycytidylic acid (pIpC). We verified efficient deletion (>95%) of *Bptf* in the BM (i.e., *Bptf*^*cKO*^) via genotyping and RT-PCR to confirm our model (Figures 1B and 1C). While *Mx1*-cre is widely used for achieving inducible gene deletion in HSPC, it is also associated with pIpC-caused interferon activation and cre-induced potential toxicity. To address these issues, we produced littermate controls with *Bptf*^*flf*^ or *Mx1-*cre alone and subjected them to pIpC administration. By fluorescence-activated cell sorting (FACS) and 4 weeks after cre induction, we observed a significantly reduced total number of lineage^−^/SCA-1^+^/c-KIT^+^ (LSK) cells and long-term (LT)-HSCs (LSK/CD150^+^/CD48^−^, Figure 1D) in the BM of *Bptf*^*cKO*^ mice, relative to controls (Figures 1D and 1E). This result shows a role for BPTF in the maintenance of primitive HSPCs, including LT-HSCs, in adult mice.

### BPTF Sustains the Self-Renewal and Repopulating Capacity of HSCs in a Cell-Autonomous Mechanism

**Figure 2:**
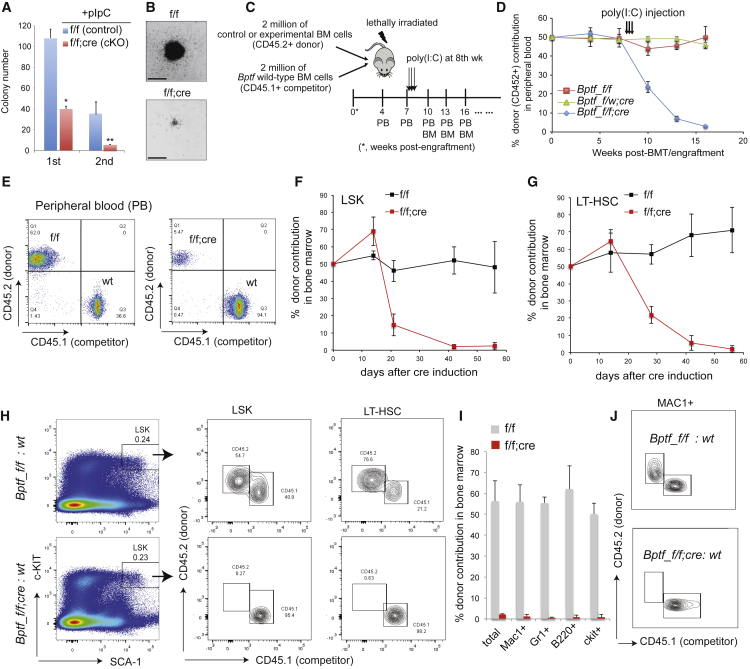
BPTF Is Essential for the Maintenance and Reconstitution Function of HSCs in a Cell-Autonomous Manner (A and B) Summary (A) and representative colony (B; scale bar, 1 mm) in colony-forming unit assays with 300 of the *Bptf*^*f/f*^ or *Bptf*^*cKO*^ (f/f; cre) LSK cells sorted 7 days after cre induction (n = 3 independent experiments; ^∗^p < 0.05; ^∗∗^p < 0.01; see also Figure S1C). (C) Outline of competitive reconstitution assay via BMT. (D) Percentage of donor-derived CD45.2^+^ cells from *Bptf*^*cKO*^ (blue; n = 8 mice) and control mice, either *Bptf*^*f/f*^ (red; n = 8) or *Bptf*^*-/w*^ (green; n = 6), in peripheral blood of recipients at the indicated time points. Error bars denote SE. (E) FACS of donor-derived CD45.2^+^ cells, either from *Bptf*^*f/f*^ or *Bptf*^*cKO*^ mice, in peripheral blood 5 weeks after cre induction. (F–H) Summary (F and G; n = 2 mice at each time point) and FACS (H) of donor-derived CD45.2^+^ cells, either from control (*Bptf*^*f/f*^) or *Bptf*^*cKO*^ mice, in the BM LSK and LT-HSC populations 8 weeks after cre induction (see also Figure S1D). (I and J) Percentage (I; n = 4 mice) and FACS (J) of donor-derived CD45.2^+^ cells from *Bptf*^*f/f*^ or *Bptf*^*cKO*^ mice in the indicated BM populations 8 weeks after cre induction (see also Figures S1E and S1F).

Next, we sought to determine whether BPTF regulates HSC function in a cell-autonomous manner. Using a colony-forming assay with sorted LSK populations, we found that *Bptf*-ablated cells produced significantly fewer and smaller colonies relative to control (Figures 2A and 2B). Similar results were seen in LSK cells with short hairpin RNA-mediated knockdown of *Bptf* (Figure S1C). We also performed competitive bone marrow transplantation (BMT) to test the reconstitution capacity of *Bptf*-null HSCs. Here, total BM cells from CD45.2^+^, *Bptf*^*f/f*^;*Mx1-*cre^+^ mice were mixed at a 1:1 ratio with wild-type competitor cells from CD45.1^+^ mice, then used as donor for BMT to lethally irradiated recipients (Figure 2C). Cells from *Bptf*^*f/f*^ or heterozygous *Bptf*^*f/w*^;*Mx1*-cre^+^ mice were used as control in BMT. When we observed stable chimerism in all cohorts 8 weeks after BMT (Figure 2D), we induced *Bptf* deletion and observed a gradual decline in the contribution of the *Bptf-*null donor cells to peripheral blood (Figures 2D and 2E). Meanwhile, the percentages of control donor cells remained stable after pIpC injection, suggesting that one *Bptf* allele is sufficient to sustain HSC function and hematopoiesis (Figures 2D and 2E).

We also examined the LSK and LT-HSC populations in recipients in the reconstitution assay (Figure S1D), and found a significantly decreased contribution of *Bptf*^*cKO*^ but not control donor cells to these primitive compartments (Figures 2F and 2G). Eight weeks after cre induction, the presence of *Bptf-*ablated donors decreased to nearly undetectable levels in HSCs and differentiated cell compartments in the BM or spleen (Figures 2H–2J, S1E, and S1F). Loss of the *Bptf*^*cKO*^ HSCs may occur through failure to maintain HSPCs' cell identity, increased apoptosis, or their combination. We assessed LSK cells 3 weeks after cre induction and did not detect a significant increase in apoptosis in *Bptf*^*cKO*^ mice relative to control (Figures S1G and S1H). Together, these results show a cell-autonomous role of BPTF in sustaining the repopulating function of HSCs.

### BPTF Activates an HSC-Specific Gene-Expression Program, Including a “Stemness” Regulatory Node that Comprises Several Master Regulators of HSCs

**Figure 3:**
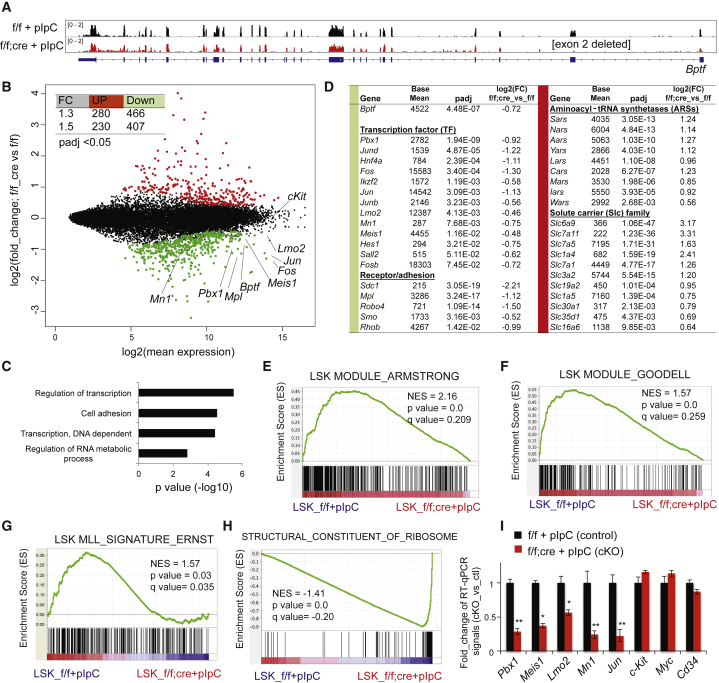
RNA-Seq Profiling Identifies a BPTF-Dependent Gene-Activation Program that Includes Several Key Master Regulators of HSCs (A) IGV view showing the RNA-seq profile of *Bptf* in the *Bptf*^*f/f*^ and *Bptf*^*cKO*^ (f/f; cre) LSK cells after pIpC treatment. For cross-sample comparison, the scale of profile is normalized with total sequencing read counts. (B) The MA plot of RNA-seq transcriptome profiles in the *Bptf*^*cKO*^ versus *Bptf*^*f/f*^ LSK cells after pIpC treatment. The x axis shows the average gene expression (log_2_-transformed) in control and knockout samples, while the y axis shows the indicated fold change by log_2_ transformation. Each dot represents a gene. The red and green colors mark genes that show significant differential expression, with a cutoff of adjusted p value (padj) < 0.05 and absolute log_2_(fold change) > 0.43. An inserted table summarizes the total number of transcripts found up- or downregulated in *Bptf*^*cKO*^ LSK cells relative to *Bptf*^*f/f*^ controls, with the indicated cutoff of fold change (FC) and padj (see also Figure S2A and Table S1). (C) GO analysis reveals the indicated gene pathway among the transcripts downregulated in *Bptf*^*cKO*^ LSK cells relative to *Bptf*^*f/f*^ controls (see also Figures S2B and S2C). (D) RNA-seq identifies genes downregulated (left; green) or upregulated (right; red) in *Bptf*^*cKO*^ LSK cells, relative to *Bptf*^*f/f*^ controls. Base Mean denotes the average RNA-seq count. (E–H) GSEA reveals enrichment of the indicated signature, either LSK “stemness” genes (E and F), a KMT2A-sustained gene network (G), or ribosomal genes (H) in *Bptf*^*cKO*^ versus *Bptf*^*f/f*^ LSK cells after cre induction (see also Figures S2D and S2E). (I) qRT-PCR using the *Bptf*^*cKO*^ versus *Bptf*^*f/f*^ LSK cells sorted on day 10 after cre induction. Data are mean ± SD (n = 3 biological replicates) and normalized to *β-actin* and *Bptf*^*f/f*^ cells. ^∗^p < 0.05; ^∗∗^p < 0.01.

To define the gene-regulatory role of BPTF in HSPCs, we performed RNA sequencing (RNA-seq) to profile transcriptomes of the LSK cells sorted from *Bptf*^*cKO*^ and *Bptf*^*f/f*^ mice 10 days after cre induction (Figure S2A). As expected, there was a lack of RNA-seq reads mapped to the *Bptf* exon 2 in *Bptf*^*cKO*^ cells due to cre-mediated deletion (Figure 3A). This produced the out-of-frame unstable *Bptf* transcripts, with reduced overall expression when compared with control (Figure 3B, *Bptf*). *Bptf*^*cKO*^ and *Bptf*^*f/f*^ LSK cells expressed comparable levels of *cKit*, an LSK marker (Figure 3B, *cKit*), and comparison of their RNA-seq profiles identified 407 downregulated and 230 upregulated transcripts due to *Bptf* ablation (with adjusted p < 0.05 and fold change > 1.5; Figure 3B [inset] and Table S1). Gene ontology (GO) and Ingenuity Pathway Analysis revealed the transcription regulation and cell adhesion-related pathways among the most downregulated ones upon BPTF loss (Figures 3C and 3D), including a TF regulatory node that consists of *Meis1*, *Pbx1*, *Mn1*, and *Lmo2* (Figure S2B). Previous studies show these TFs as master regulators of HSC by establishing the gene-regulatory circuits essential for HSC self-renewal and identity (Heuser et al., 2011, Wang et al., 2005, Wilson et al., 2010). Consistently, when we related our RNA-seq data to the previously reported HSC gene sets by gene set enrichment analysis (GSEA), we found that, relative to *Bptf*^*cKO*^, *Bptf*^*f/f*^ LSK cells are enriched with LSK signature genes (Chambers et al., 2007, Krivtsov et al., 2006) and those sustained by a crucial HSC regulator, KMT2A (Artinger et al., 2013) (Figures 3E–3G). Also, the AP1 complex TFs (e.g., *Fos* and *Jun*) showed decreased expression in *Bptf*^*cKO*^ LSK cells (Figure 3D, left). GO and GSEA also found the biosynthesis- and translation-related pathways among the most upregulated ones in *Bptf*^*cKO*^ cells (Figures 3H and S2C–S2E), a phenomenon similar to that reported in the *KMT2A-*null HSPCs (Artinger et al., 2013). The most upregulated genes include many aminoacyl-tRNA synthetase and solute carrier protein genes (Figure 3D, right). By RT-PCR, we validated downregulation of *Pbx1*, *Meis1*, *Mn1*, and *Lmo2* upon *Bptf* loss in LSK cells, while expression of *Myc*, *cKit*, and *Cd34* was unchanged (Figure 3I). Thus, we identified a BPTF-dependent gene-expression program that includes several master TFs of HSCs, which supports a role of BPTF in defining HSCs' cellular identity.

### BPTF Potentiates DNA Accessibility at the HSC “Stemness” Genes

**Figure 4:**
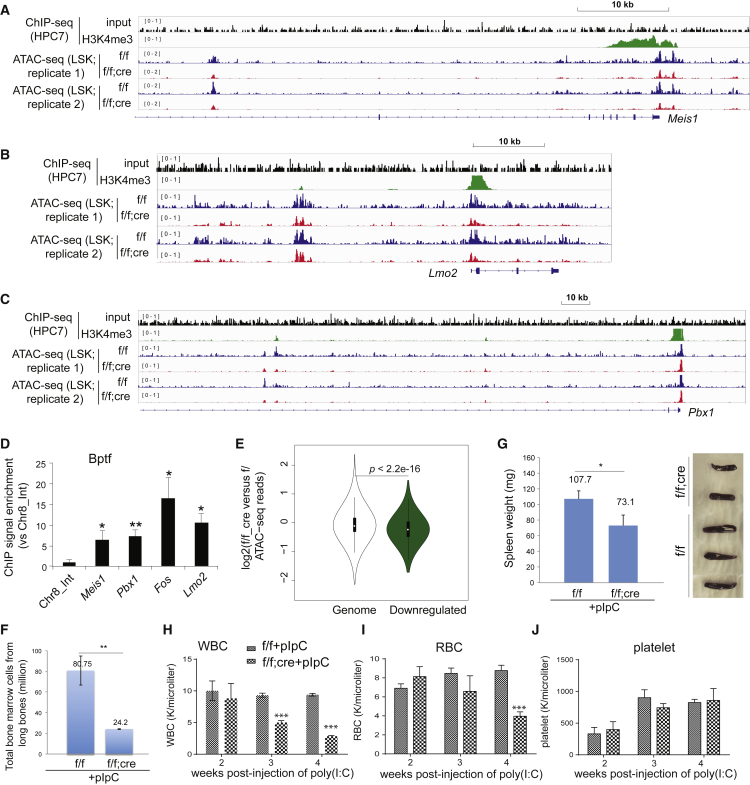
BPTF Potentiates Chromatin Accessibility at HSC “Stemness” Genes (A–C) ChIP-seq profiles of H3K4me3 and input at the indicated genes in HPC-7 cells, and their ATAC-seq profiles in *Bptf*^*f/f*^ versus *Bptf*^*cKO*^ LSK cells 7 days after cre induction. For cross-sample comparison, the scales of profiles are normalized with total sequencing read counts (see also Figure S3A). (D) BPTF ChIP at the indicated gene promoter in HPC-7 cells. Fold of enrichment in signals, shown as mean ± SD (n = 3 biological replicates), was normalized to input and to a control locus (Chr8_Int). ^∗^p < 0.05; ^∗∗^p < 0.01. (E) Comparison of ATAC-seq data in *Bptf*^*cKO*^ versus *Bptf*^*f/f*^ LSK cells shows a significant reduction of ATAC-seq signals at the promoters of genes showing downregulation due to BPTF loss, relative to genome background. Plotted at y axis are log_2_-transformed ratios of promoter-associated ATAC-seq reads between two samples, either at all genes (left) or at the top 500 downregulated genes in *Bptf*^*cKO*^ LSK cells (right), relative to *Bptf*^*f/f*^ control (see also Figures S3B and S3C). (F and G) Total cell numbers in the femur (F) and the size of spleen (G) in *Bptf*^*f/f*^ (n = 3) versus *Bptf*^*cKO*^ (n = 4) mice 4 weeks after cre induction. ^∗^p < 0.05; ^∗∗^p < 0.01. (H–J) Complete blood counts of peripheral blood collected from *Bptf*^*cKO*^ mice (n = 4) and *Bptf*^*f/f*^ littermates (n = 4): WBC, white blood cells (H); RBC, red blood cells (I); and platelets (J) (see also Figure S4). ^∗∗∗^p < 0.001.

To test whether BPTF directly targets the “stemness” genes identified by RNA-seq, we assessed BPTF binding by chromatin immunoprecipitation (ChIP). We used HPC-7 cells because ChIP requires large cell numbers, which prevents the use of primary HSPCs, and HPC-7 cells were previously used as an HSPC mimic to map the genomic binding of HSC regulators (Wilson et al., 2010). ChIP-sequencing (ChIP-seq) analysis revealed high H3K4me3 at the promoters of “stemness” genes such as *Meis1*, *Pbx1*, and *Lmo2* (Figures 4A–4C and S3A, top panel), providing a putative platform for BPTF binding. Unfortunately, BPTF ChIP-seq failed due to inadequate pull-down of DNA, but conventional ChIP-qPCR showed significant binding of BPTF to the tested promoter loci at “stemness” genes, compared with the negative control (Figure 4D). Because BPTF/NURF modulates nucleosomal positioning, we also used the assay for transposase-accessible chromatin followed by sequencing (ATAC-seq) to measure DNA accessibility in *Bptf*^*cKO*^ versus *Bptf*^*f/f*^ LSK cells after cre induction (Figure S3B). Upon *Bptf* ablation, we did not see a dramatic change in global ATAC-seq signals (Figure S3C) but observed significantly reduced DNA accessibility at the promoters of downregulated genes (Figure 4E) such as *Meis1*, *Pbx1*, *Fos*, and *Lmo2* (Figures 4A–4C and S3A). We also observed decreased ATAC-seq signals at putative distal or intragenic enhancers of these TF genes (Figures 4A–4C and S3A). Together, these genomic data support crucial roles of BPTF in potentiating DNA accessibility and appropriate expression of key HSC TF genes. Further work is needed to firmly define genomic binding of BPTF in HSPCs.

### Hematopoietic-Specific Loss of BPTF Leads to Bone Marrow Failure, Anemia, and Leukopenia

Given the impaired function of *Bptf*^*cKO*^ HSCs, we predicted that lineage-committed populations in *Bptf*^*cKO*^ mice would be affected. Four weeks after *Bptf* deletion, the bones from *Bptf*^*cKO*^ mice appeared pale and showed significant decrease in the total BM cell number when compared with control (Figure 4F). Moreover, we observed that the *Bptf*^*cKO*^ mice possessed smaller spleens relative to control, which suggests a defect in splenic B cell development (Figure 4G). FACS of total splenic cells confirmed our observation, with a significant reduction in the B220^+^ cells (Figure S4A). Furthermore, complete blood counts revealed anemia, leukopenia, and granulocytopenia in *Bptf*^*cKO*^ mice, phenotypes that arise from the dysfunctional repopulation capacity of HSCs (Figures 4H–4J and S4B–S4D). Thus, we show *Bptf* to be essential for normal hematopoiesis.

## Discussion

### BPTF Plays an Essential Role in the Maintenance and Functionality of HSCs

How adult stem cells sustain themselves remains as an intriguing question. Using knockout and reconstitution systems, we showed an essential requirement of BPTF for maintaining the HSPC populations and their repopulating capacity. Mechanistically, our transcriptome profiling revealed a previously unappreciated, BPTF-dependent gene-activation program, which includes a set of master TFs known to be vital for HSC self-renewal (*Meis1*, *Pbx1*, *Mn1*, and *Lmo2*), the AP1 complex, and the MLL/KMT2A signature genes. BPTF also sustains an open chromatin state at target “stemness” genes. Thus, BPTF acts as a safeguard of adult hematopoiesis, ensuring HSCs' reconstitution function. In support, BPTF loss caused BM failure phenotypes that are reminiscent of what was observed for *KMT2A*-null HSCs (Artinger et al., 2013, Jude et al., 2007). BPTF appears to be more crucial for blood formation under stressed conditions (e.g., reconstitution in irradiated mice) than in the steady state, a phenomenon also described in a conditional *KMT2A*-null model (McMahon et al., 2007). However, depth study is required to dissect the potentially overlapping and distinctive roles for BPTF and KMT2 in HSC self-renewal and blood formation.

### BPTF Controls Vital Gene-Expression Programs to Sustain Homeostasis of Multiple Cell Lineages

In related research, BPTF acts as a crucial regulator of mammary gland and epidermal stem cells (Frey et al., 2017, Mulder et al., 2012), melanocytes (Koludrovic et al., 2015), and T cells (Landry et al., 2011, Wu et al., 2016). Here, an intriguing question is how the general chromatin regulator BPTF controls a defined yet distinct gene-expression program among different cell lineages. Presumably these cells differ in patterns of histone modifications, which can stabilize BPTF binding to genes essential for lineage definition. Also, BPTF/NURF interacts with DNA-binding factors such as CTCF (Qiu et al., 2015) and c-MYC (Richart et al., 2016a). A multivalent interaction of NURF to histone modifications, TFs, and other recruiting factors can act in concert to dictate distinct genomic targeting of BPTF/NURF.

### The Essential Function of BPTF in Normal Tissue Raises a Concern on Targeting It in Cancer Therapy

Recently, the oncogenic role of BPTF was reported in melanoma (Dar et al., 2015, Dar et al., 2016), pancreatic tumors, and Burkitt's lymphoma (Richart et al., 2016b), where BPTF was shown to promote the gene program related to tumor cell growth or survival such as c-MYC and BCL2. Bptf carries an H3K4me3-binding PHD and an acetyl-histone-binding bromodomain. Both motifs including PHD associate with human disease (Baker et al., 2008, Gough et al., 2014, Wang et al., 2009) and can be potentially druggable (Arrowsmith et al., 2012). BPTF was proposed as a drug target for cancer therapy (Richart et al., 2016a). However, increasing evidence now shows a vital requirement of BPTF for normal homeostasis of a range of tissues. Such broad homeostatic function for Bptf requires additional studies to address toxicity associated with targeting this protein in cancer.

## Experimental Procedures

Details of additional procedures such as BMT, FACS, sorting, ATAC-seq, and ChIP are provided in Supplemental Experimental Procedures.

### Knockout Mice

CD45.2^+^ B/6 mice carrying the *Bptf*^f/f^ allele (stock #009367) or *Mx1*-cre were purchased from the Jackson Laboratory and crossed to produce *Bptf*^*f/f*^;*Mx1*-cre mice and control littermates. To induce *Bptf* knockout in the BM, we injected 2- to 3-month-old mice with pIpC (Sigma) three times every other day. UNC-Chapel Hill Institutional Animal Care and Use Committee approved all animal experiments.

### RNA-Seq

RNA was extracted from sorted LSK cells with the picoRNA Kit (Applied Biosystems) and the RNA-seq library was prepared with Illumina kits according to the manufacturer’s protocol, followed by deep sequencing.

## Author Contributions

G.G.W. designed the project. B.X., L.C., J.M.B., X.L., D.F.A., R.L., S.R., and G.G.W. performed experiments and interpreted data. J.S.P., D.Z., and D.C. analyzed genomic data. G.G.W. wrote the manuscript, and D.F.A. and B.X. critically read the paper.

## Accession Numbers

The genomics data are deposited to the NCBI GEO repository under GEO: GSE108441.

## Supplemental Information

Document S1. Supplemental Experimental Procedures, Figures S1–S4, and Table S2

Table S1. RNA-Seq Reveals Differentially Expressed Genes in Bptf-cKO versus Control (f/f) LSK Cells, Related to Figure 3

Document S2. Article plus Supplemental Information
