# Supplementary material for: The Chromatin Remodeler BPTF Activates a Stemness Gene-Expression Program Essential for the Maintenance of Adult Hematopoietic Stem Cells
Source: Stem Cell Reports. 2018 Feb 15;10(3):675–83. doi: 10.1016/j.stemcr.2018.01.020 (PMC5918338; doi:10.1016/j.stemcr.2018.01.020)
Supplement: Document S1. Supplemental Experimental Procedures, Figures S1–S4, and Table S2 [file mmc1.pdf]

Stem Cell Reports, Volume *10*

Supplemental Information

The Chromatin Remodeler BPTF Activates a Stemness Gene-Expression Program Essential for the Maintenance of Adult Hematopoietic Stem Cells

Bowen Xu, Ling Cai, Jason M. Butler, Dongliang Chen, Xiongdong Lu, David F. Allison, Rui Lu, Shahin Rafii, Joel S. Parker, Deyou Zheng, and Gang Greg Wang

Supplemental Information

The chromatin remodeler BPTF activates a stemness gene-expression program essential for the maintenance of adult hematopoietic stem cells

Bowen Xu et al.

Inventory of Supplemental Information

Supplemental Figures S1-S4 and Legends

- Figure S1 related to main Fig 1 and 2
- Figure S2 related to main Fig 3
- Figure S3-4 related to main Fig 4
- Table S1 related to main Fig 3
- Table S2 related to main Fig 1, 3 and 4

Supplemental Experimental Procedures

Reference

Figure S1

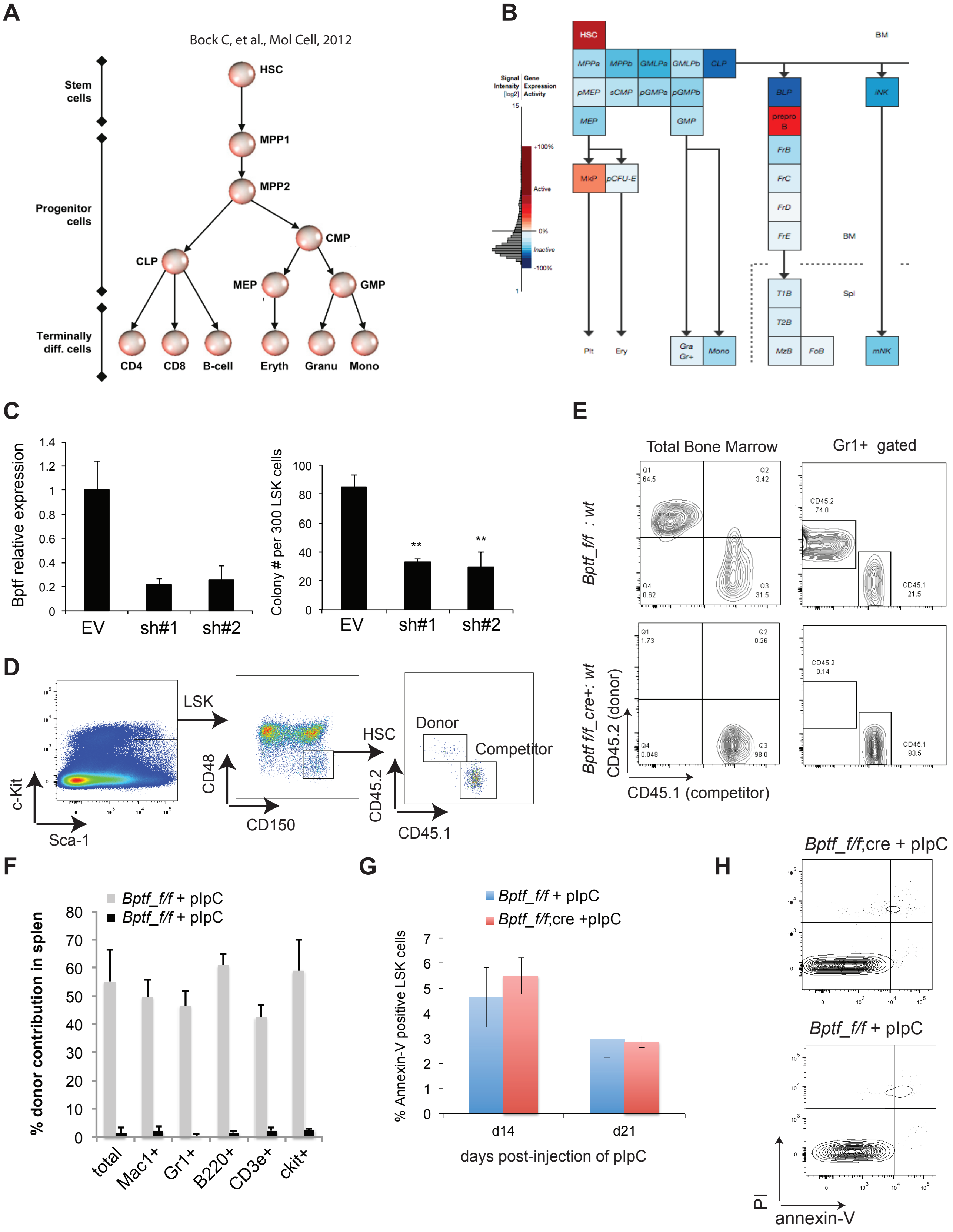
A
Bock C, et al., Mol Cell, 2012
Stem cells
Progenitor cells
Terminally diff. cells
HSC
MPP1
MPP2
CLP
CMP
MEP
GMP
CD4
CD8
B-cell
Eryth
Granu
Mono
B
HSC
MPPa
MPPb
GMLPa
GMLPb
CLP
pMEP
sCMP
pGMPa
pGMPb
MEP
GMP
MkP
pCFU-E
BLP
prepro B
FrB
FrC
FrD
FrE
T1B
T2B
MzB
FoB
iNK
mNK
Plt
Ery
Gra Gr+
Mono
BM
Spl
C
Bptf relative expression
EV
sh#1
sh#2
Colony # per 300 LSK cells
D
LSK
HSC
Donor
Competitor
c-Kit
Sca-1
CD48
CD150
CD45.2
CD45.1
E
Total Bone Marrow
Gr1+ gated
Bptf_f/f : wt
Bptf f/f_cre+: wt
CD45.2 (donor)
CD45.1 (competitor)
F
Bptf_f/f + pIpC
Bptf_f/f + pIpC
% donor contribution in splen
total
Mac1+
Gr1+
B220+
CD3e+
ckit+
G
Bptf_f/f + pIpC
Bptf_f/f;cre +pIpC
% Annexin-V positive LSK cells
d14
d21
days post-injection of pIpC
H
Bptf_f/f;cre + pIpC
Bptf_f/f + pIpC
PI
annexin-V

Figure S1. Maintenance of the HSPC compartments and their reconstitution capacities requires *Bptf* expression.

(A) Hematopoietic cell subtypes shown in main Figure 1A, with their expression dataset obtained from a published study (Bock et al., 2012). **(B)** *Bptf* expression (microarray probe: 1427311_at) during hematopoietic development according to the “Gene Expression Commons” dataset (Seita et al., 2012). **(C)** Effect of *Bptf* knockdown by either of the two validated shRNA (left panel) on colony formation (right). Plotted in the right panel were mean ± SD (n= 3 biological replicates) of CFU colonies derived from 300 of the LSK cells stably transduced with empty vector control (EV) or shRNA. Statistical analysis was determined by two-tailed Student’s t-test: *, $p<0.05$; **, $p<0.01$. **(D)** A representative FACS scheme detecting either the wild-type competitor-derived CD45.1+ cells or the donor-derived CD45.2+ cells. Shown are $Bptf^{f/f}$;*Mx1*-cre+ ($Bptf^{cKO}$) donor-derived cells analyzed five weeks after cre induction among the LSK and LT-HSC (LSK/CD150+/CD48-) subpopulations in the BM. **(E)** Typical FACS plots of the donor-derived CD45.2+ cells, either from $Bptf^{f/f}$ or $Bptf^{cKO}$ mice, in the indicated BM cells eight weeks after cre induction. **(F)** Summary of average percentages of the donor-derived CD45.2+ cells, either from the control $Bptf^{f/f}$ (n=4) or $Bptf^{cKO}$ (n=4) mice, among the indicated spleen cells eight weeks after cre induction. **(G-H)** Summary (panel **G;** n=3 mice) and FACS plot (panel **H**) of the annexin-V+ apoptotic cells among the LSK populations at the indicated time point after cre induction.

Figure S2

A

Summary of LSK cell RNA-seq read tags

Sample	Replicate	Total # of reads
f/f+plpC	1	36,604,132
	2	20,226,077
f/f;cre+plpC	1	44,658,576
	2	20,573,687

C

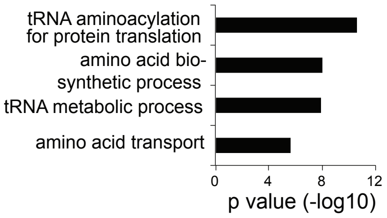

B

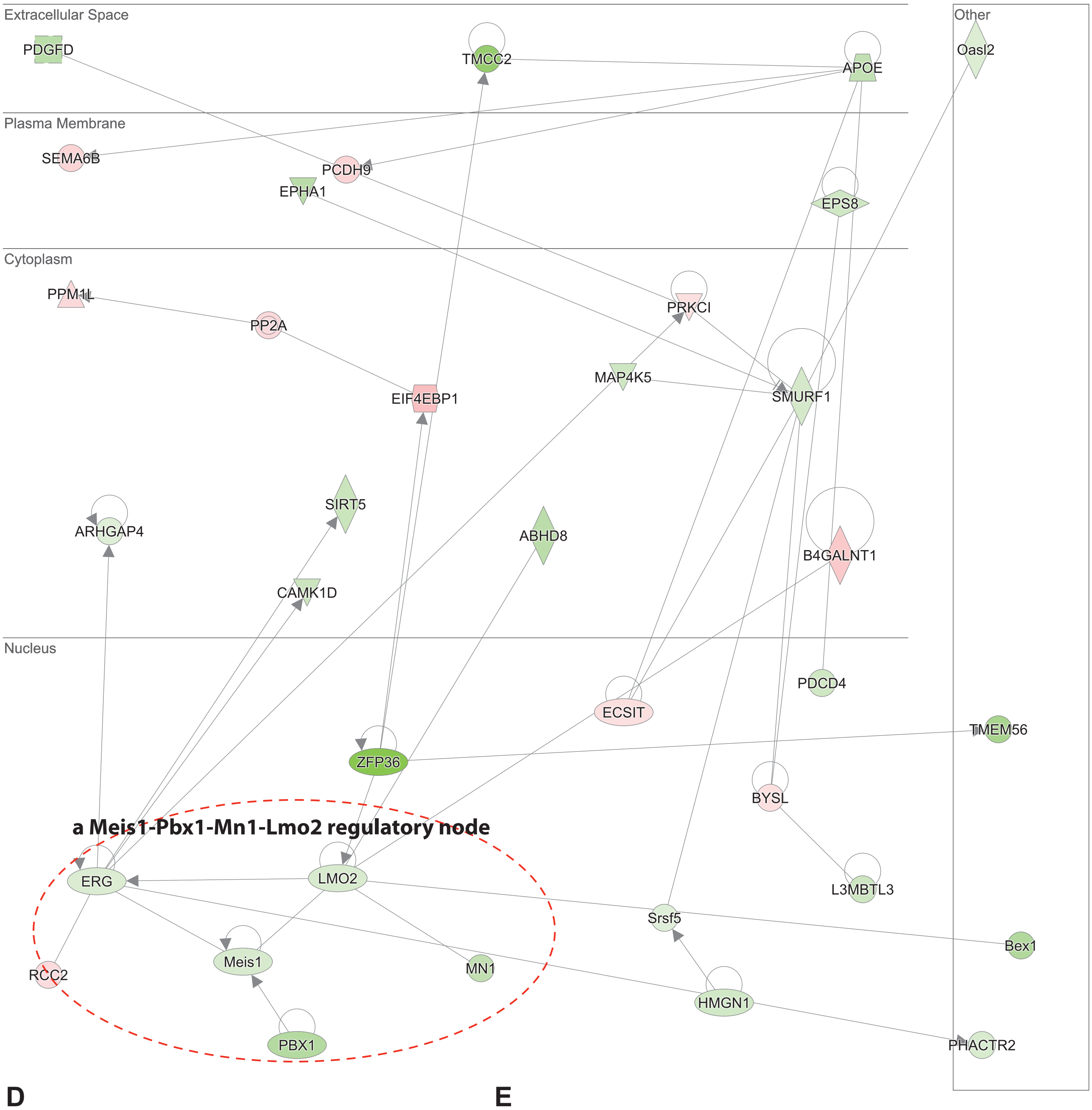

D

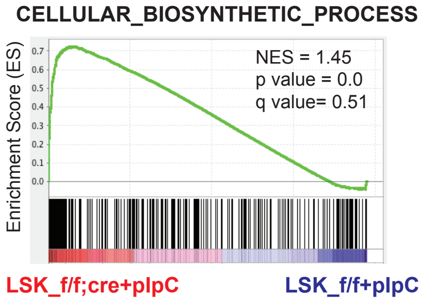

E

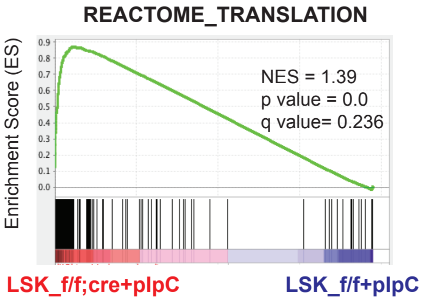

Figure S2. RNA-seq profiling identifies an unappreciated, BPTF-dependent transcriptional activation program that includes a crucial 'stemess' regulatory node consisting of several HSC master regulators.

(A) Summary of read counts from RNA-sequencing (RNA-seq) profiles of the control (*Bptf*$^{f/f}$) and *Bptf*^{cKO} (*Bptf*$^{f/f}$*;Mx1*-cre) LSK cells that were sorted from 5 mice (in two biological replicates) on day 10 after cre induction.

(B) Analysis of the deregulated genes with the Ingenuity Pathway Analysis (IPA) tool identifies the significant perturbation (p<10E-47) of the shown molecular pathway that is related to cancer and development. Green and red show the down- and up-regulated genes, respectively, in the *Bptf*^{cKO} LSK cells relative to *Bptf*$^{f/f}$ controls, with genes categorized according to their subcellular localizations.

(C) GO analysis reveals the indicated gene pathways among the transcripts found up-regulated in the *Bptf*^{cKO} LSK cells relative to *Bptf*$^{f/f}$ controls.

(D-E) GSEA reveals enrichment of the indicated gene signature in the *Bptf*$^{f/f}$ versus *Bptf*^{cKO} LSK cells isolated from mice after cre induction.

Figure S3

A

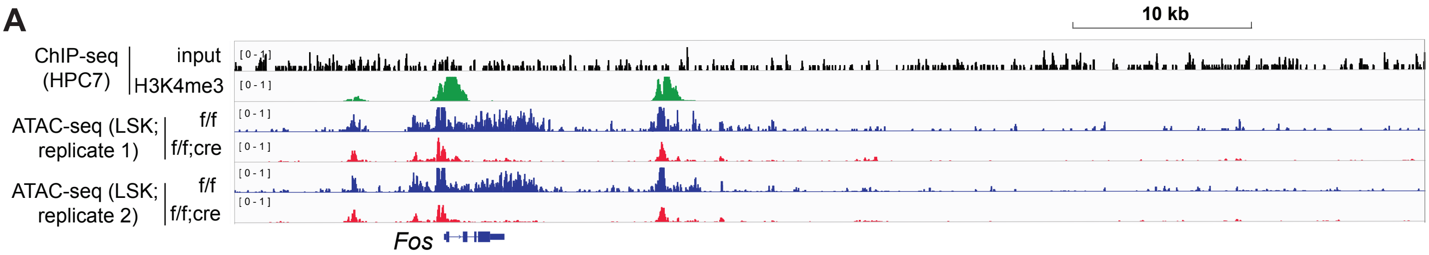

B

Summary of LSK cell ATAC-seq read tags

	Cell types	File ID	Total # of Reads
replicate #1	LSK control (Bptf_f/f)	ATAC-BPWT_150225	10,024,904
	LSK Bptf_cKO	ATAC-BPKO_150225	21,874,128
replicate #2	LSK control (Bptf_f/f)	ATAC-BPWT_150319	16,582,933
	LSK Bptf_cKO	ATAC-BPKO_150319	37,729,978
replicate #3	LSK control (Bptf_f/f)	ATAC-BPWT_150130	8,441,275
	LSK Bptf_cKO	ATAC-BPKO_150130	18,397,932

C

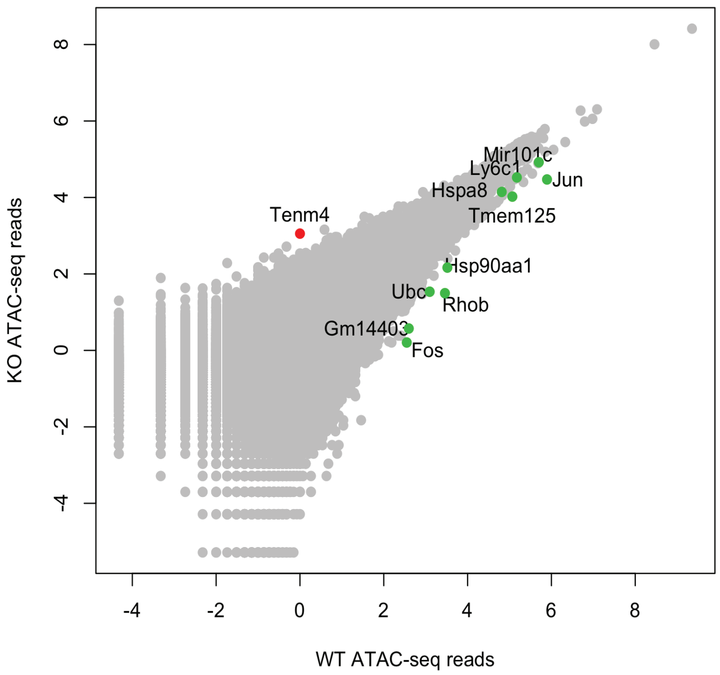

Figure S3. BPTF potentiates chromatin accessibility of key HSC ‘stemess’ TF genes.

(A) ChIP-seq profiles showing H3K4me3 and the chromatin inputs at the indicated gene in HPC-7 cells, as well as the ATAC-seq profiles among the control versus *Bptf*^{cKO} LSK cells that were sorted on day 7 after cre induction. For cross-sample comparison, the scales of profiles are normalized with total sequencing read counts.

(B) Summary of read counts from ATAC-sequencing (ATAC-seq) profiles of the control (*Bptf*$^{f/f}$) and *Bptf*^{cKO} (*Bptf*$^{f/f}$*;Mx1*-cre) LSK cells on day 7 after cre induction.

(C) Comparison of global DNA accessibility among the *Bptf*^{cKO} (y-axis) versus *Bptf*$^{f/f}$ (x-axis) LSK cells on day 7 after cre induction. Plotted at x- and y-axis are ATAC-seq read counts for each called individual peak in log2 transformation of per million sequencing depth.

Figure S4

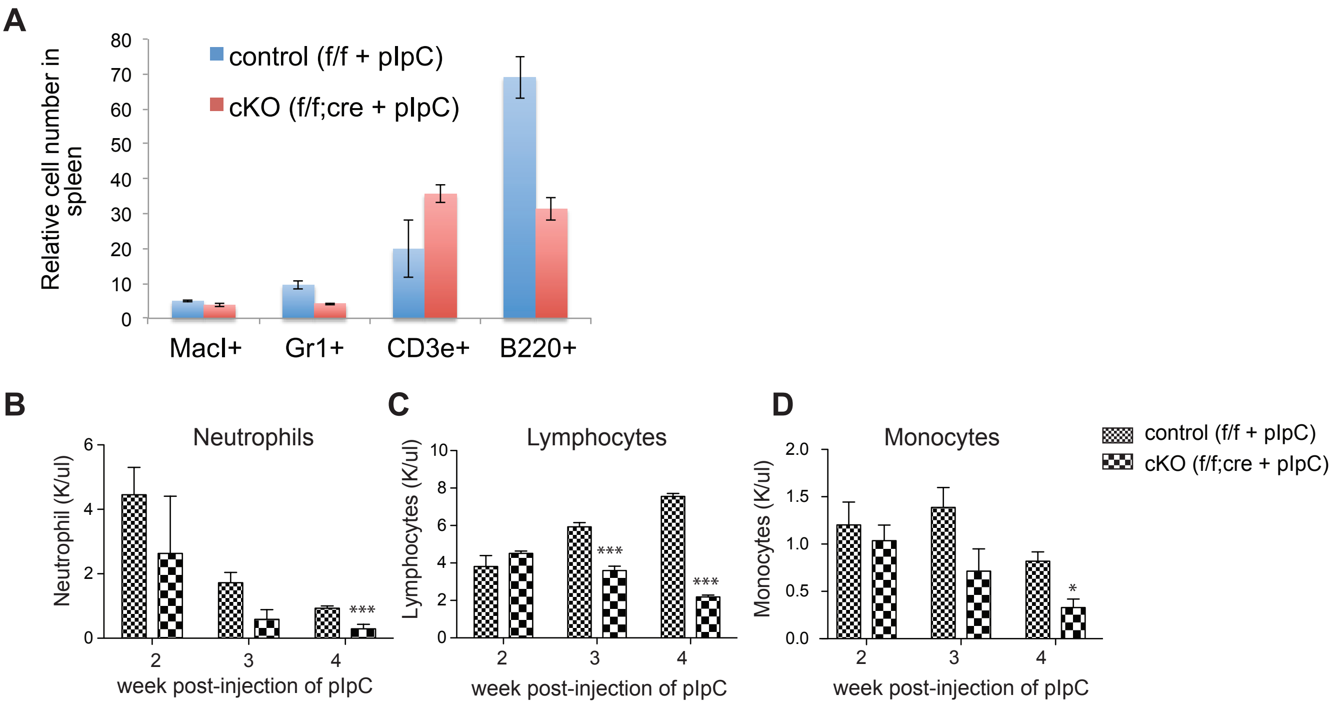
A
Relative cell number in spleen
control (f/f + pIpC)
cKO (f/f;cre + pIpC)
MacI+
Gr1+
CD3e+
B220+
B
Neutrophils
Neutrophil (K/ul)
week post-injection of pIpC
C
Lymphocytes
Lymphocytes (K/ul)
week post-injection of pIpC
D
Monocytes
Monocytes (K/ul)
week post-injection of pIpC
control (f/f + pIpC)
cKO (f/f;cre + pIpC)

Figure S4. *Bptf* loss in the hematopoietic compartment causes severe defects in the reconstitution capacities of HSPCs, anemia and leukopenia.

(A) Summary of averaged cell numbers of the indicated splenic cell populations isolated from the control (*Bptf*$^{f/f}$, n=3) and *Bptf*^{cKO} (n=4) mice, as analyzed by FACS.

(B-D) Complete blood cell counts of peripheral blood collected from the *Bptf*^{cKO} mice and control (*Bptf*$^{f/f}$) littermates (n=4 mice) at the indicated time after cre induction.

Table S2. Primer information. Related to Figure 1, 3 and 4.

	Gene	Forward	Reverse	Note
**RT-qPCR**				
	Bptf	agcagcttcaggagccatag	aggggtgggatgtctttttc	target exon-2 to check deletion efficiency of BPTF cKO
		cgttcgacacgaacctattg	gctaactggacctttgtgctg	
	Fos (c-Fos)	TTTCAACGCCGACTACGAGG	GCGCAAAAGTCCTGTGTGTT	
	Jun	TGGGCACATCACCACTACAC	TCTGGCTATGCAGTTCAGCC	
	Pbx1	GCGCCGGGAGCCCATTTCTGC	GGTCCCTCCGGCCCCATCCTG	
	Meis1	AAGGTGATGGCTTGGACAAC	TGTGCCAACTGCTTTTTCTG	
	Lmo2	CTGGACCCGTCTGAGGAAC	GTCACAGCTGAGGCAATCCT	
	Hoxa5	GCAAGCTGCACATTAGTCAC	GCATGAGCTATTTCGATCCT	
	Hoxa9	ACAATGCCGAGAATGAGAGC	CAGCGTCTGGTGTTTTGTGT	
	Cd34	CTGGAATCCGAGAAGTGAGG	GCCTCCTCCTTTTCACACAG	
	cKit	ATGAACAGGACCTCGGCTAAC	ACTGGCATCAGAGTTGGACAC	
	Hprt1	TCCTCCTCAGACCGCTTTT	CCTGGTTCATCATCGCTAATC	internal control
	beta-Actin	ACCAACTGGGACGACATGGA	GGTCTCAAACATGATCTGGGTCAT	internal control
	Gapdh	TGCACCACCAACTGCTTAGC	GGCATGGACTGTGGTCATGAG	internal control
**ChIP-qPCR**				
	Meis1	gcagttgcaaagagggagag	gcccgctttccttgaatc	near TSS
	Pbx1	CTCCTCCTCCTCCTCTCAGG	CTCTTTCCTCCTCCGTGTCTC	near TSS
	Fos	AATCCTACACGCGGAAGGTC	CCCCGTCTTGGCATACATCT	near TSS
	Chr8_int	AAGGGGCCTCTGCTTAAAAA	AGAGCTCCATGGCAGGTAGA	an intergenic region of chr 8 as negative control

Supplemental Methods

Mouse. Genotyping information for *Bptf*$^{f/f}$ and *Mx1*-cre alleles is available from the Jackson Laboratory. CD45.1+ B/6 congenic mice (B6-LY5.2/Cr) were purchased from NCI Frederick Mouse Repository. UNC-Chapel Hill Institutional Animal Care and Use Committee approved all animal experiments.

Bone marrow transplantation (BMT) and in vivo competitive reconstitution assay. Total BM cells isolated from femur and tibia of donor (CD45.2+) and competitor (CD45.1+) b/6 mice were treated with the ACK lysis buffer (0.15 M NH4Cl, 10 mM KHCO3, 0.1 mM EDTA [pH 7.2]) to remove red blood cells. An equal amount mixture of unfractionated donor and competitor BM cells was transplanted via tail veil injection into the lethally irradiated, congenic CD45.1+ b/6 recipient mice (irradiated with a single dose of 1,000 rads; carried out by the Animal Studies Core affiliated to UNC Cancer Center). Stable engraftment of donor (CD45.2+) and competitor (CD45.1+) BM cells was monitored by FACS analysis of the peripheral blood (PB) cells. Briefly, about 20ul of PB was collected from tail veil nick, subject to ACK lysis, and stained with antibodies of CD45.2-APC (ebioscience, cat# 17-0454) and CD45.1-FITC (ebioscience, cat# 11-0453). To induce gene deletion, the experimental BMT cohort (i.e. engrafted with *Bptf*$^{f/f}$*;Mx1-cre* donor BM cells) and control cohorts (i.e. engrafted with *Bptf*$^{f/f}$ alone or the heterozygous *Bptf*$^{f/w}$*;Mx1*-cre donor BM cells) were subject to poly(I:C) injection as described before (Li et al., 2011). The percentages of donor(CD45.2+)-derived and competitor(CD45.1+)-derived cells were analyzed with FACS in the PB or BM. For FACS of LSK cells, about 10 million total BM cells were harvested and stained with FITC-labeled antibody cocktail for lineage markers (CD11b-FITC [eBioscience 11-0112-82], Gr1-FITC [eBioscience 11-5931-82], CD3e-FITC [eBioscience 11-0031-82],

Ter119-FITC [eBioscience 11-5921-82], B220-FITC [eBioscience 11-0452-82], CD8a-FITC [eBioscience 11-0081-82], CD5-FITC [eBioscience 11-0051-81], CD2-FITC [eBioscience 11-0021-81], CD4-FITC [eBioscience 11-0042-82]), cKIT-APC (BD Biosciences 561074), SCA1-PE-Cy7 (BD Biosciences 558162), CD150-PE (eBioscience 12-1502), CD48-PerCP-Cy5.5 (BioLegend 103422), CD45.2-Alexa Fluor 700 (eBioscience 56-0454-82), and CD45.1-APC-eFluor 780 (eBioscience 47-0453-82). Samples were analyzed with a LSRII machine (BD Biosciences; UNC flow core).

Flow cytometry (FACS) analysis and cell sorting. Total BM cells were isolated from the femur and tibia of mice. FACS-based analysis of lineage-/SCA-1+/cKIT+ (LSK) cells and long-term hematopoietic stem cells (LT-HSC) were carried out using LSRII (BD Biosciences; UNC flow core) as previously described (Poulos et al., 2016; Xie et al., 2014). For sorting of LSK cells, total BM cells harvested from the femur, tibia and pelvis of 5 mice were combined and subject to c-KIT-APC+ selection (Miltenyi biotec). The obtained c-Kit positive selected cells are incubated with a lineage marker cocktail consisting of B220-FITC, CD11b-FITC, Gr1-FITC, CD3e-FITC, Ter119-FITC, CD8a-FITC, CD5-FITC, CD2-FITC and CD4-FITC (purchased from eBioscience as listed above), as well as the SCA-1-PE-Cy7 antibodies (BD Biosciences 558162), for 30 minutes. Sorting of LSK cells was carried out by the UNC flow core with a FACSAria II machine. For assaying apoptosis, the cells were incubated with Annexin-V FITC (Cell Signaling #6592) and analyzed as described before (Xie et al., 2014; Xu et al., 2015).

RNA-sequencing (RNA-seq) and data analysis. RNA-seq raw data were first trimmed to remove the Illumina adapters by using a custom script (UNC HTSF/Genomics core and UNC Cancer Center Bioinformatics core), and then mapped to the mouse reference genome using the HISAT2 with default parameters (Kim et al., 2015). Gene expression

levels were quantified with featureCounts (Liao et al., 2014) with default parameters that discarded multi-mapping reads. Raw gene counts were normalized, and differential expression calculated using DESeq2 with “betaPrior” turned on (Love et al., 2014). The MA plot of gene expression data was generated using the DESeq2 table and R.

Cells. HPC-7 cells, a cell line mimicking HSPCs (a kind gift of Prof. Leif Carlsson, Umeå University, Sweden), were cultured and maintained as previously described (Dahl et al., 2008; Wilson et al., 2010).

ChIP-sequencing (ChIP-seq). ChIP-seq was carried out as described before (Lu et al., 2016; Xu et al., 2015). The H3K4me3 antibody used for ChIP-seq was purchased from Abcam (ab8580).

ATAC-seq. ATAC-seq was carried out with the Epicentre Tn5 transposome and Illuminia kit according to the manufacturer protocol.

ATAC-seq data analysis. ATAC-seq reads were aligned to the mouse genome (mm9) using the software BWA (version “0.7.15-r1140”) and default parameters (Li and Durbin, 2010). The software MACS2 (Feng et al., 2012) was used for calling peaks from the individual replicates, with the “--nomodel --extsize 150 –q 0.1” option. The read counts within the called peaks were used for comparison across samples.

Gene Set Enrichment Analysis (GSEA), Gene Ontology (GO) analysis, and Ingenuity Pathway Analysis (IPA). DESeq2 normalized counts were used for GSEA by the javaGSEA program (Subramanian et al., 2005) with default parameters. Briefly, GSEA with the Molecular Signature Database as well as customized gene sets was carried out according to providers’ instructions as described before (Lu et al., 2016; Xu et al., 2015). GO was carried out as previously described (Lu et al., 2016; Xu et al., 2015). For IPA, the deregulated genes identified by RNA-seq were uploaded and

analyzed using the online IPA tool (www.qiagenbioinformatics.com) to determine the perturbed molecular networks.

RT-qPCR and ChIP-qPCR. RT-qPCR and ChIP-qPCR was carried out to examine the gene expression changes and genomic binding, respectively, as described before (Lu et al., 2016; Xu et al., 2015). Primer information was provided in supplemental table S2. BPTF antibodies (Bethyl #A300-973 and Millipore #ABE24) were used in ChIP assays.

Colony formation unit (CFU) assay. 300 of LSK cells freshly sorted from BM were mixed with the methylcellulose medium (StemCell Technologies; MethoCult GF M3434) and plated in triplicate as carried out before (Lu et al., 2016; Xu et al., 2015). For replating, approximately 5,000 cells isolated from cell colonies in the previous plating were seeded again in the same semi-solid medium. CFUs were counted every 10 days post-plating. Identity of each colony was defined by morphology according to manufacturer's specifications (StemCell Technologies).

shRNA and gene knockdown. The pLKO lentiviral constructs with the *Bptf* specific shRNA were purchased from Sigma (see below tables) and validated in our previous work (Zhou et al., 2016). HEK293 cells were used for producing virus as previously described (Lu et al., 2016; Xu et al., 2015).

sh_#1	TRCN0000238661	ACCGGATTATTACGGTGTTAT	mouse	CDS
sh_#2	TRCN0000238660	GACCGTAACCTCGTTCATATT	mouse	3UTR

Statistics. Data are presented as the mean ± SD of at least three independent experiments. Statistical analysis was performed with two-tailed Student's t test for comparing two sets of data with assumed normal distribution.

<u>Reference of supplement document:</u>

Bock, C., Beerman, I., Lien, W.H., Smith, Z.D., Gu, H., Boyle, P., Gnirke, A., Fuchs, E., Rossi, D.J., and Meissner, A. (2012). DNA methylation dynamics during in vivo differentiation of blood and skin stem cells. Molecular cell *47*, 633-647.
Dahl, L., Richter, K., Hagglund, A.C., and Carlsson, L. (2008). Lhx2 expression promotes self-renewal of a distinct multipotential hematopoietic progenitor cell in embryonic stem cell-derived embryoid bodies. PLoS ONE *3*, e2025.
Feng, J., Liu, T., Qin, B., Zhang, Y., and Liu, X.S. (2012). Identifying ChIP-seq enrichment using MACS. Nature protocols *7*, 1728-1740.
Kim, D., Langmead, B., and Salzberg, S.L. (2015). HISAT: a fast spliced aligner with low memory requirements. Nat Methods *12*, 357-360.
Li, H., and Durbin, R. (2010). Fast and accurate long-read alignment with Burrows-Wheeler transform. Bioinformatics *26*, 589-595.
Li, L., Jothi, R., Cui, K., Lee, J.Y., Cohen, T., Gorivodsky, M., Tzchori, I., Zhao, Y., Hayes, S.M., Bresnick, E.H.*, et al.* (2011). Nuclear adaptor Ldb1 regulates a transcriptional program essential for the maintenance of hematopoietic stem cells. Nature immunology *12*, 129-136.
Liao, Y., Smyth, G.K., and Shi, W. (2014). featureCounts: an efficient general purpose program for assigning sequence reads to genomic features. Bioinformatics *30*, 923-930.
Love, M.I., Huber, W., and Anders, S. (2014). Moderated estimation of fold change and dispersion for RNA-seq data with DESeq2. Genome Biol *15*, 550.
Lu, R., Wang, P., Parton, T., Zhou, Y., Chrysovergis, K., Rockowitz, S., Chen, W.Y., Abdel-Wahab, O., Wade, P.A., Zheng, D.*, et al.* (2016). Epigenetic Perturbations by Arg882-Mutated DNMT3A Potentiate Aberrant Stem Cell Gene-Expression Program and Acute Leukemia Development. Cancer cell *30*, 92-107.
Poulos, M.G., Ramalingam, P., Gutkin, M.C., Kleppe, M., Ginsberg, M., Crowley, M.J., Elemento, O., Levine, R.L., Rafii, S., Kitajewski, J.*, et al.* (2016). Endothelial-specific inhibition of NF-kappaB enhances functional haematopoiesis. Nat Commun *7*, 13829.
Seita, J., Sahoo, D., Rossi, D.J., Bhattacharya, D., Serwold, T., Inlay, M.A., Ehrlich, L.I., Fathman, J.W., Dill, D.L., and Weissman, I.L. (2012). Gene Expression Commons: an open platform for absolute gene expression profiling. PLoS ONE *7*, e40321.
Subramanian, A., Tamayo, P., Mootha, V.K., Mukherjee, S., Ebert, B.L., Gillette, M.A., Paulovich, A., Pomeroy, S.L., Golub, T.R., Lander, E.S.*, et al.* (2005). Gene set enrichment analysis: a knowledge-based approach for interpreting genome-wide expression profiles. Proceedings of the National Academy of Sciences of the United States of America *102*, 15545-15550.
Wilson, N.K., Foster, S.D., Wang, X., Knezevic, K., Schutte, J., Kaimakis, P., Chilarska, P.M., Kinston, S., Ouwehand, W.H., Dzierzak, E.*, et al.* (2010). Combinatorial transcriptional control in blood stem/progenitor cells: genome-wide analysis of ten major transcriptional regulators. Cell stem cell *7*, 532-544.
Xie, H., Xu, J., Hsu, J.H., Nguyen, M., Fujiwara, Y., Peng, C., and Orkin, S.H. (2014). Polycomb repressive complex 2 regulates normal hematopoietic stem cell function in a developmental-stage-specific manner. Cell stem cell *14*, 68-80.
Xu, B., On, D.M., Ma, A., Parton, T., Konze, K.D., Pattenden, S.G., Allison, D.F., Cai, L., Rockowitz, S., Liu, S.*, et al.* (2015). Selective inhibition of EZH2 and EZH1 enzymatic activity by a small molecule suppresses MLL-rearranged leukemia. Blood *125*, 346-357.
Zhou, Y., Wang, L., Vaseghi, H.R., Liu, Z., Lu, R., Alimohamadi, S., Yin, C., Fu, J.D., Wang, G.G., Liu, J.*, et al.* (2016). Bmi1 Is a Key Epigenetic Barrier to Direct Cardiac Reprogramming. Cell stem cell *18*, 382-395.
