# Supplementary material for: The Chromatin Remodeler BPTF Activates a Stemness Gene-Expression Program Essential for the Maintenance of Adult Hematopoietic Stem Cells
Source: Stem Cell Reports. 2018 Feb 15;10(3):675–83. doi: 10.1016/j.stemcr.2018.01.020 (PMC5918338; doi:10.1016/j.stemcr.2018.01.020)
Supplement: Document S2. Article plus Supplemental Information [file mmc3.pdf]

Stem Cell Reports
Report
OPEN ACCESS

The Chromatin Remodeler BPTF Activates a Stemness Gene-Expression Program Essential for the Maintenance of Adult Hematopoietic Stem Cells

Bowen Xu,[1,2] Ling Cai,[1,2] Jason M. Butler,[3] Dongliang Chen,[1,2] Xiongdong Lu,[4] David F. Allison,[1,2] Rui Lu,[1,2] Shahin Rafii,[3] Joel S. Parker,[1] Deyou Zheng,[5] and Gang Greg Wang[1,2,*]
[1]Lineberger Comprehensive Cancer Center
[2]Department of Biochemistry and Biophysics
University of North Carolina at Chapel Hill School of Medicine, Chapel Hill, NC 27599, USA
[3]Department of Medicine and Ansary Stem Cell Institute, Weill Cornell Medical College, New York, NY 10065, USA
[4]Laboratory of Biochemistry and Molecular Biology, Rockefeller University, New York, NY 10065, USA
[5]Department of Neuroscience and Neurology, Albert Einstein College of Medicine, Bronx, NY 10461, USA
*Correspondence: greg_wang@med.unc.edu
https://doi.org/10.1016/j.stemcr.2018.01.020

SUMMARY

Self-renewal and differentiation of adult stem cells are tightly regulated partly through configuration of chromatin structure by chromatin remodelers. Using knockout mice, we here demonstrate that bromodomain PHD finger transcription factor (BPTF), a component of the nucleosome remodeling factor (NURF) chromatin-remodeling complex, is essential for maintaining the population size of hematopoietic stem/progenitor cells (HSPCs), including long-term hematopoietic stem cells (HSCs). *Bptf*-deficient HSCs are defective in reconstituted hematopoiesis, and hematopoietic-specific knockout of *Bptf* caused profound defects including bone marrow failure and anemia. Genome-wide transcriptome profiling revealed that BPTF loss caused downregulation of HSC-specific gene-expression programs, which contain several master transcription factors (*Meis1*, *Pbx1*, *Mn1*, and *Lmo2*) required for HSC maintenance and self-renewal. Furthermore, we show that BPTF potentiates the chromatin accessibility of key HSC "stemness" genes. These results demonstrate an essential requirement of the chromatin remodeler BPTF and NURF for activation of "stemness" gene-expression programs and proper function of adult HSCs.

INTRODUCTION

Appropriate self-renewal and differentiation of adult stem cells are essential for tissue homeostasis and are tightly controlled by various cellular and molecular mechanisms, including the dynamic regulation of chromatin structure by ATP-dependent chromatin-remodeling complexes (Kadoch and Crabtree, 2015; Wang et al., 2007). These remodelers use energy produced from ATP hydrolysis to configure nucleosomal positioning and modulate DNA accessibility. Such a process ensures fidelity of crucial gene-expression programs during developmental processes such as lineage specification. In support of this pathway contributing to cell fate determination, somatic mutation of chromatin remodeler genes is common in human disease, including cancer (Kadoch and Crabtree, 2015; Wang et al., 2007).

Bromodomain PHD finger transcription factor (BPTF) is a core and largest component of the conserved, multisubunit nucleosome remodeling factor (NURF) complex (Landry et al., 2008; Ruthenburg et al., 2011). NURF loosens condensed chromatin to promote DNA accessibility and target gene activation (Ruthenburg et al., 2011; Schwanbeck et al., 2004; Wysocka et al., 2006). While global knockout of *Bptf* in mice leads to lethality on embryonic day 8.5, demonstrating its requirement for early development (Landry et al., 2008), clinical studies reveal loss-of-function mutation of *BPTF* in individuals with syndromic neurodevelopmental anomalies (Stankiewicz et al., 2017). Furthermore, BPTF was recently shown to be critical for the maintenance or differentiation of mammary gland stem cells (Frey et al., 2017), melanocytes (Dar et al., 2016; Large et al., 2016), and T cells (Landry et al., 2011; Wu et al., 2016). BPTF contains two motifs in its C terminus, a PHD finger and a bromodomain that bind to histone H3 lysine 4 trimethylation (H3K4me3) and histone acetylation, respectively (Chi et al., 2010; Ruthenburg et al., 2011; Wysocka et al., 2006). Deposition of these two modifications occurs partly via the histone methyltransferase MLL/KMT2A and associated histone acetyltransferases (Dou et al., 2005). While previous works detail the essential role for KMT2A in regulation of hematopoietic and neuronal stem cells (Artinger et al., 2013; Jude et al., 2007; Lim et al., 2009), the specific contributions of BPTF remain undefined in this process.

Using knockout mice, we here show BPTF as a crucial chromatin regulator of hematopoietic stem cells (HSCs). Reconstitution assays demonstrate that *Bptf*-null HSCs exhibited the decreased repopulating capacity, causing severe hematopoietic defects. Our genomic profiling shows that ablation of BPTF in hematopoietic stem/progenitor cells (HSPCs) leads to decreased expression of an HSC-specific gene-expression program, which includes a master transcription factor (TF) regulatory node (*Meis1*, *Pbx1*, *Mn1*, and *Lmo2*) known to be crucial for HSC self-renewal and

This is an open access article under the CC BY license (http://creativecommons.org/licenses/by/4.0/).

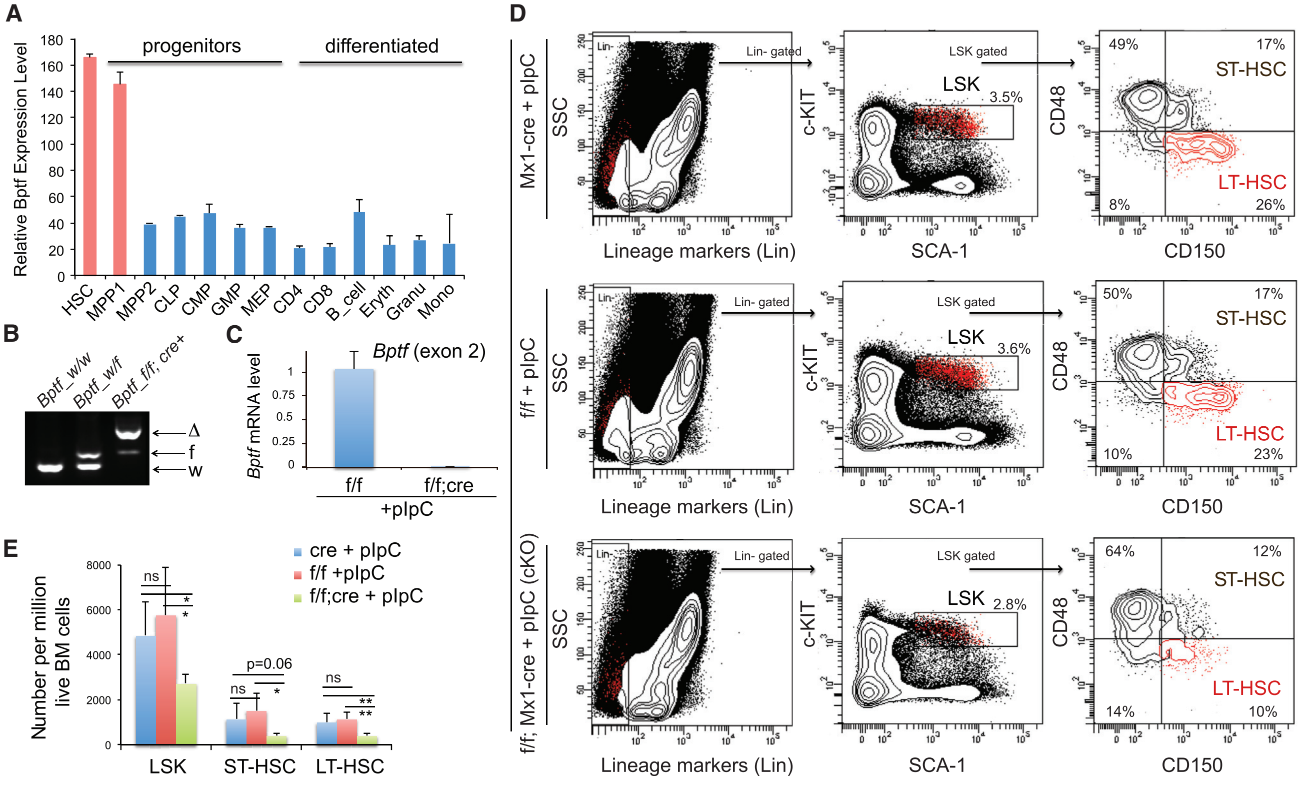

Figure 1. Maintenance of Adult HSPCs Including LT-HSC Requires BPTF

(A) *Bptf* expression in hematopoiesis (see also Figures S1A and S1B).

(B and C) Genotyping (B) and RT-PCR (C; n = 3 biological replicates) confirm deletion of the *Bptf* exon 2 in total bone marrow (BM) 1 week after cre induction. w, wild-type; f, floxed; Δ, deleted (*Bptf^{cKO}*).

(D and E) FACS (D) and summary (E) of percentages of the LSK and LT-HSC cells in the BM, 4 weeks after cre induction in the *Bptf^{cKO}* (f/f; cre, n = 5 mice) or control littermates with *Bptf$^{f/f}$* (f/f) or *Mx1*-cre (cre) alone (n = 4 mice). Numbers in (D) indicate the percentage of gated cells. Plots in (E) are mean ± SD, with statistical analysis defined by two-tailed Student's t test: ns, not significant; *p < 0.05; **p < 0.01.

function. We also find that BPTF potentiates the chromatin accessibility of these HSC TF genes. Collectively, our results support a vital requirement of the BPTF chromatin remodeler for the maintenance of adult HSPCs and for the activation of a gene transcription program essential for HSC functions.

RESULTS

Maintenance of Adult HSPCs, Including Long-Term HSCs, Requires *Bptf* Expression

Using transcriptome datasets of hematopoiesis (Bock et al., 2012; Seita et al., 2012), we found *Bptf* preferentially expressed in the primitive HSPC compartment (Figures 1A, S1A, and S1B). To study the role of BPTF in HSPCs, we produced inducible knockout mice (*Bptf$^{f/f}$*;*Mx1*-cre) designed to ablate *Bptf* from the bone marrow (BM) upon activation of *Mx1*-cre by polyinosinic-polycytidylic acid (pIpC). We verified efficient deletion (>95%) of *Bptf* in the BM (i.e., *Bptf^{cKO}*) via genotyping and RT-PCR to confirm our model (Figures 1B and 1C). While *Mx1*-cre is widely used for achieving inducible gene deletion in HSPC, it is also associated with pIpC-caused interferon activation and cre-induced potential toxicity. To address these issues, we produced littermate controls with *Bptf$^{f/f}$* or *Mx1*-cre alone and subjected them to pIpC administration. By fluorescence-activated cell sorting (FACS) and 4 weeks after cre induction, we observed a significantly reduced total number of lineage$^-$/SCA-1$^+$/c-KIT$^+$ (LSK) cells and long-term (LT)-HSCs (LSK/CD150$^+$/CD48$^-$, Figure 1D) in the BM of *Bptf^{cKO}* mice, relative to controls (Figures 1D and 1E). This result shows a role for BPTF in the maintenance of primitive HSPCs, including LT-HSCs, in adult mice.

BPTF Sustains the Self-Renewal and Repopulating Capacity of HSCs in a Cell-Autonomous Mechanism

Next, we sought to determine whether BPTF regulates HSC function in a cell-autonomous manner. Using a

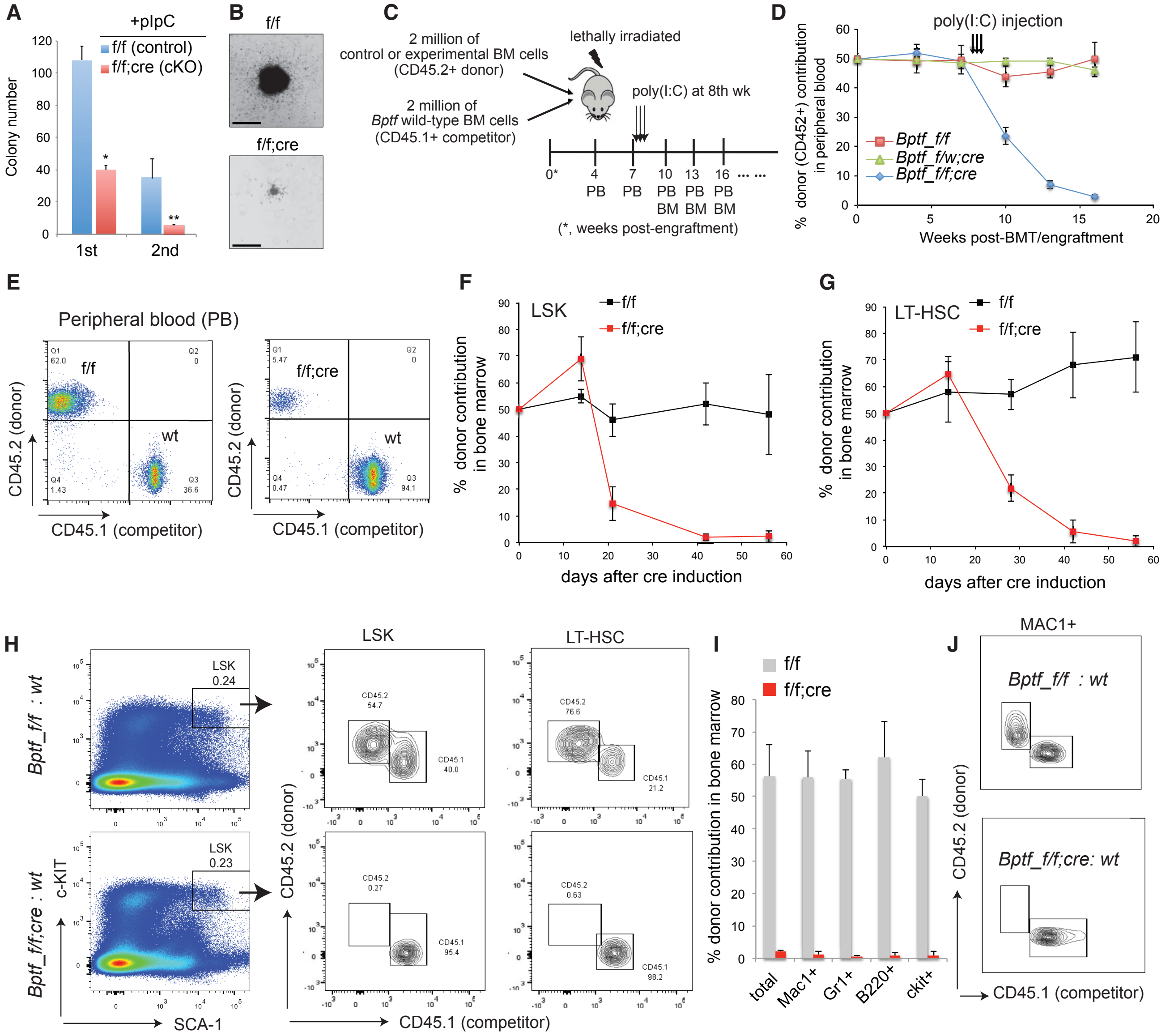

Figure 2. BPTF Is Essential for the Maintenance and Reconstitution Function of HSCs in a Cell-Autonomous Manner

(A and B) Summary (A) and representative colony (B; scale bar, 1 mm) in colony-forming unit assays with 300 of the *Bptf*$^{f/f}$ or *Bptf*^{cKO} (f/f; cre) LSK cells sorted 7 days after cre induction (n = 3 independent experiments; *p < 0.05; **p < 0.01; see also Figure S1C).

(C) Outline of competitive reconstitution assay via BMT.

(D) Percentage of donor-derived CD45.2$^+$ cells from *Bptf*^{cKO} (blue; n = 8 mice) and control mice, either *Bptf*$^{f/f}$ (red; n = 8) or *Bptf*$^{f/w}$ (green; n = 6), in peripheral blood of recipients at the indicated time points. Error bars denote SE.

(E) FACS of donor-derived CD45.2$^+$ cells, either from *Bptf*$^{f/f}$ or *Bptf*^{cKO} mice, in peripheral blood 5 weeks after cre induction.

(F–H) Summary (F and G; n = 2 mice at each time point) and FACS (H) of donor-derived CD45.2$^+$ cells, either from control (*Bptf*$^{f/f}$) or *Bptf*^{cKO} mice, in the BM LSK and LT-HSC populations 8 weeks after cre induction (see also Figure S1D).

(I and J) Percentage (I; n = 4 mice) and FACS (J) of donor-derived CD45.2$^+$ cells from *Bptf*$^{f/f}$ or *Bptf*^{cKO} mice in the indicated BM populations 8 weeks after cre induction (see also Figures S1E and S1F).

colony-forming assay with sorted LSK populations, we found that *Bptf*-ablated cells produced significantly fewer and smaller colonies relative to control (Figures 2A and 2B). Similar results were seen in LSK cells with short hairpin RNA-mediated knockdown of *Bptf* (Figure S1C). We also performed competitive bone marrow transplantation (BMT) to test the reconstitution capacity of *Bptf*-null HSCs. Here, total BM cells from CD45.2$^+$, *Bptf*$^{f/f}$;*Mx1*-cre$^+$ mice were

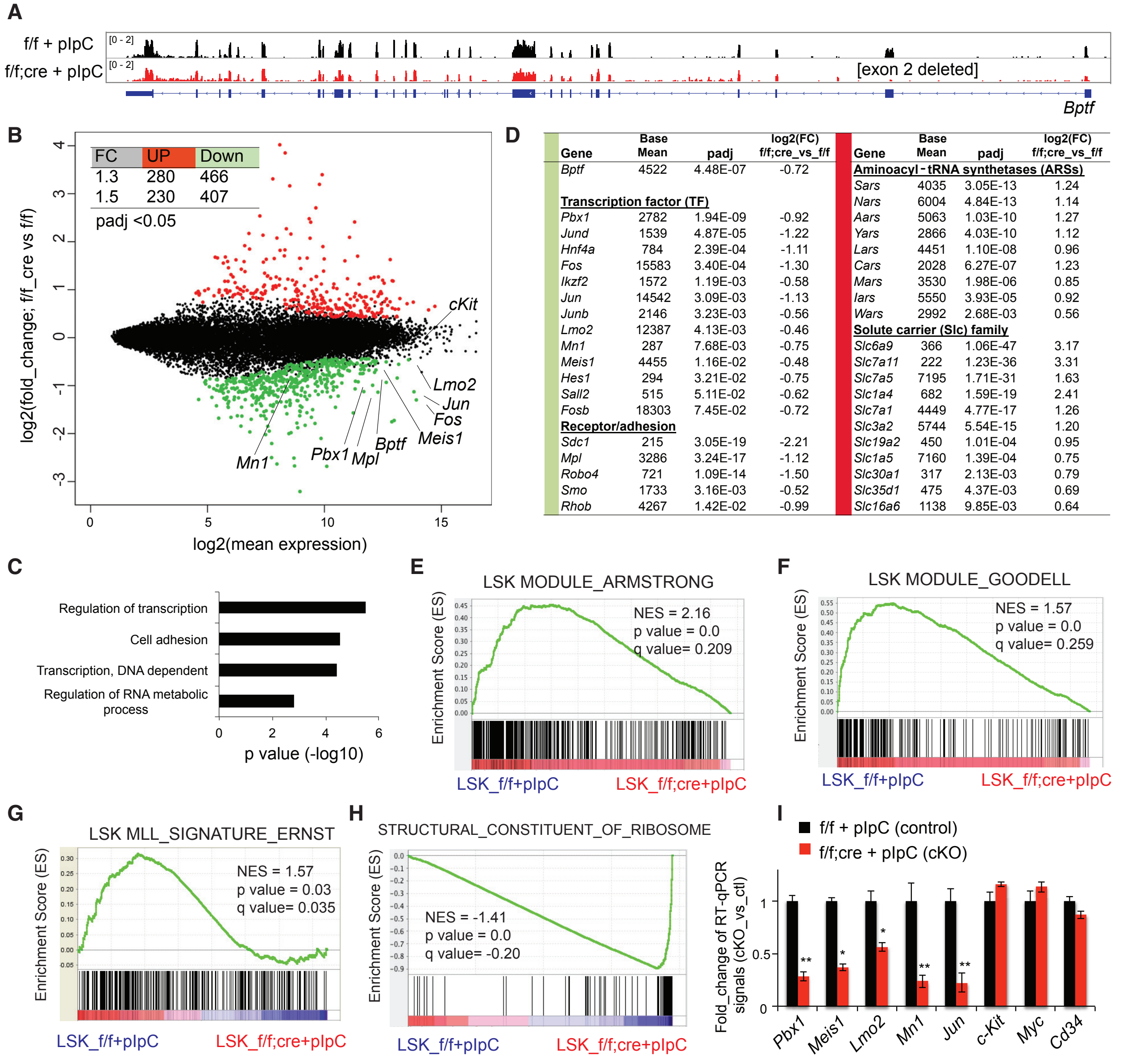

Gene	Base Mean	padj	log2(FC) f/f;cre_vs_f/f
Bptf	4522	4.48E-07	-0.72
**Transcription factor (TF)**			
Pbx1	2782	1.94E-09	-0.92
Jund	1539	4.87E-05	-1.22
Hnf4a	784	2.39E-04	-1.11
Fos	15583	3.40E-04	-1.30
Ikzf2	1572	1.19E-03	-0.58
Jun	14542	3.09E-03	-1.13
Junb	2146	3.23E-03	-0.56
Lmo2	12387	4.13E-03	-0.46
Mn1	287	7.68E-03	-0.75
Meis1	4455	1.16E-02	-0.48
Hes1	294	3.21E-02	-0.75
Sall2	515	5.11E-02	-0.62
Fosb	18303	7.45E-02	-0.72
**Receptor/adhesion**			
Sdc1	215	3.05E-19	-2.21
Mpl	3286	3.24E-17	-1.12
Robo4	721	1.09E-14	-1.50
Smo	1733	3.16E-03	-0.52
Rhob	4267	1.42E-02	-0.99

Gene	Base Mean	padj	log2(FC) f/f;cre_vs_f/f
**Aminoacyl-tRNA synthetases (ARSs)**			
Sars	4035	3.05E-13	1.24
Nars	6004	4.84E-13	1.14
Aars	5063	1.03E-10	1.27
Yars	2866	4.03E-10	1.12
Lars	4451	1.10E-08	0.96
Cars	2028	6.27E-07	1.23
Mars	3530	1.98E-06	0.85
Iars	5550	3.93E-05	0.92
Wars	2992	2.68E-03	0.56
**Solute carrier (Slc) family**			
Slc6a9	366	1.06E-47	3.17
Slc7a11	222	1.23E-36	3.31
Slc7a5	7195	1.71E-31	1.63
Slc1a4	682	1.59E-19	2.41
Slc7a1	4449	4.77E-17	1.26
Slc3a2	5744	5.54E-15	1.20
Slc19a2	450	1.01E-04	0.95
Slc1a5	7160	1.39E-04	0.75
Slc30a1	317	2.13E-03	0.79
Slc35d1	475	4.37E-03	0.69
Slc16a6	1138	9.85E-03	0.64

Figure 3. RNA-Seq Profiling Identifies a BPTF-Dependent Gene-Activation Program that Includes Several Key Master Regulators of HSCs

(A) IGV view showing the RNA-seq profile of *Bptf* in the $Bptf^{f/f}$ and $Bptf^{cKO}$ (f/f; cre) LSK cells after pIpC treatment. For cross-sample comparison, the scale of profile is normalized with total sequencing read counts.

(B) The MA plot of RNA-seq transcriptome profiles in the $Bptf^{cKO}$ versus $Bptf^{f/f}$ LSK cells after pIpC treatment. The x axis shows the average gene expression (log_2-transformed) in control and knockout samples, while the y axis shows the indicated fold change by log_2 transformation. Each dot represents a gene. The red and green colors mark genes that show significant differential expression, with a cutoff of adjusted p value (padj) < 0.05 and absolute log_2(fold change) > 0.43. An inserted table summarizes the total number of transcripts found up- or downregulated in $Bptf^{cKO}$ LSK cells relative to $Bptf^{f/f}$ controls, with the indicated cutoff of fold change (FC) and padj (see also Figure S2A and Table S1).

(C) GO analysis reveals the indicated gene pathway among the transcripts downregulated in $Bptf^{cKO}$ LSK cells relative to $Bptf^{f/f}$ controls (see also Figures S2B and S2C).

(D) RNA-seq identifies genes downregulated (left; green) or upregulated (right; red) in $Bptf^{cKO}$ LSK cells, relative to $Bptf^{f/f}$ controls. Base Mean denotes the average RNA-seq count.

(legend continued on next page)

mixed at a 1:1 ratio with wild-type competitor cells from CD45.1^{+} mice, then used as donor for BMT to lethally irradiated recipients (Figure 2C). Cells from *Bptf*$^{f/f}$ or heterozygous *Bptf*$^{f/w}$;*Mx1*-cre^{+} mice were used as control in BMT. When we observed stable chimerism in all cohorts 8 weeks after BMT (Figure 2D), we induced *Bptf* deletion and observed a gradual decline in the contribution of the *Bptf*-null donor cells to peripheral blood (Figures 2D and 2E). Meanwhile, the percentages of control donor cells remained stable after pIpC injection, suggesting that one *Bptf* allele is sufficient to sustain HSC function and hematopoiesis (Figures 2D and 2E).

We also examined the LSK and LT-HSC populations in recipients in the reconstitution assay (Figure S1D), and found a significantly decreased contribution of *Bptf*^{cKO} but not control donor cells to these primitive compartments (Figures 2F and 2G). Eight weeks after cre induction, the presence of *Bptf*-ablated donors decreased to nearly undetectable levels in HSCs and differentiated cell compartments in the BM or spleen (Figures 2H–2J, S1E, and S1F). Loss of the *Bptf*^{cKO} HSCs may occur through failure to maintain HSPCs' cell identity, increased apoptosis, or their combination. We assessed LSK cells 3 weeks after cre induction and did not detect a significant increase in apoptosis in *Bptf*^{cKO} mice relative to control (Figures S1G and S1H). Together, these results show a cell-autonomous role of BPTF in sustaining the repopulating function of HSCs.

BPTF Activates an HSC-Specific Gene-Expression Program, Including a "Stemness" Regulatory Node that Comprises Several Master Regulators of HSCs

To define the gene-regulatory role of BPTF in HSPCs, we performed RNA sequencing (RNA-seq) to profile transcriptomes of the LSK cells sorted from *Bptf*^{cKO} and *Bptf*$^{f/f}$ mice 10 days after cre induction (Figure S2A). As expected, there was a lack of RNA-seq reads mapped to the *Bptf* exon 2 in *Bptf*^{cKO} cells due to cre-mediated deletion (Figure 3A). This produced the out-of-frame unstable *Bptf* transcripts, with reduced overall expression when compared with control (Figure 3B, *Bptf*). *Bptf*^{cKO} and *Bptf*$^{f/f}$ LSK cells expressed comparable levels of *cKit*, an LSK marker (Figure 3B, *cKit*), and comparison of their RNA-seq profiles identified 407 downregulated and 230 upregulated transcripts due to *Bptf* ablation (with adjusted $p < 0.05$ and fold change > 1.5; Figure 3B [inset] and Table S1). Gene ontology (GO) and Ingenuity Pathway Analysis revealed the transcription regulation and cell adhesion-related pathways among the most downregulated ones upon BPTF loss (Figures 3C and 3D), including a TF regulatory node that consists of *Meis1*, *Pbx1*, *Mn1*, and *Lmo2* (Figure S2B). Previous studies show these TFs as master regulators of HSC by establishing the gene-regulatory circuits essential for HSC self-renewal and identity (Heuser et al., 2011; Wang et al., 2005; Wilson et al., 2010). Consistently, when we related our RNA-seq data to the previously reported HSC gene sets by gene set enrichment analysis (GSEA), we found that, relative to *Bptf*^{cKO}, *Bptf*$^{f/f}$ LSK cells are enriched with LSK signature genes (Chambers et al., 2007; Krivtsov et al., 2006) and those sustained by a crucial HSC regulator, KMT2A (Artinger et al., 2013) (Figures 3E–3G). Also, the AP1 complex TFs (e.g., *Fos* and *Jun*) showed decreased expression in *Bptf*^{cKO} LSK cells (Figure 3D, left). GO and GSEA also found the biosynthesis- and translation-related pathways among the most upregulated ones in *Bptf*^{cKO} cells (Figures 3H and S2C–S2E), a phenomenon similar to that reported in the *KMT2A*-null HSPCs (Artinger et al., 2013). The most upregulated genes include many aminoacyl-tRNA synthetase and solute carrier protein genes (Figure 3D, right). By RT-PCR, we validated downregulation of *Pbx1*, *Meis1*, *Mn1*, and *Lmo2* upon *Bptf* loss in LSK cells, while expression of *Myc*, *cKit*, and *Cd34* was unchanged (Figure 3I). Thus, we identified a BPTF-dependent gene-expression program that includes several master TFs of HSCs, which supports a role of BPTF in defining HSCs' cellular identity.

BPTF Potentiates DNA Accessibility at the HSC "Stemness" Genes

To test whether BPTF directly targets the "stemness" genes identified by RNA-seq, we assessed BPTF binding by chromatin immunoprecipitation (ChIP). We used HPC-7 cells because ChIP requires large cell numbers, which prevents the use of primary HSPCs, and HPC-7 cells were previously used as an HSPC mimic to map the genomic binding of HSC regulators (Wilson et al., 2010). ChIP-sequencing (ChIP-seq) analysis revealed high H3K4me3 at the promoters of "stemness" genes such as *Meis1*, *Pbx1*, and *Lmo2* (Figures 4A–4C and S3A, top panel), providing a putative platform for BPTF binding. Unfortunately, BPTF ChIP-seq failed due to inadequate pull-down of DNA, but conventional ChIP-qPCR showed significant binding of BPTF to the tested promoter loci at "stemness" genes, compared with the negative control (Figure 4D). Because BPTF/NURF modulates nucleosomal positioning, we also used the assay for transposase-accessible chromatin followed by sequencing (ATAC-seq) to measure DNA accessibility in *Bptf*^{cKO} versus *Bptf*$^{f/f}$ LSK cells after

(E–H) GSEA reveals enrichment of the indicated signature, either LSK "stemness" genes (E and F), a KMT2A-sustained gene network (G), or ribosomal genes (H) in *Bptf*^{cKO} versus *Bptf*$^{f/f}$ LSK cells after cre induction (see also Figures S2D and S2E).
(I) qRT-PCR using the *Bptf*^{cKO} versus *Bptf*$^{f/f}$ LSK cells sorted on day 10 after cre induction. Data are mean ± SD (n = 3 biological replicates) and normalized to *β-actin* and *Bptf*$^{f/f}$ cells. *p < 0.05; **p < 0.01.

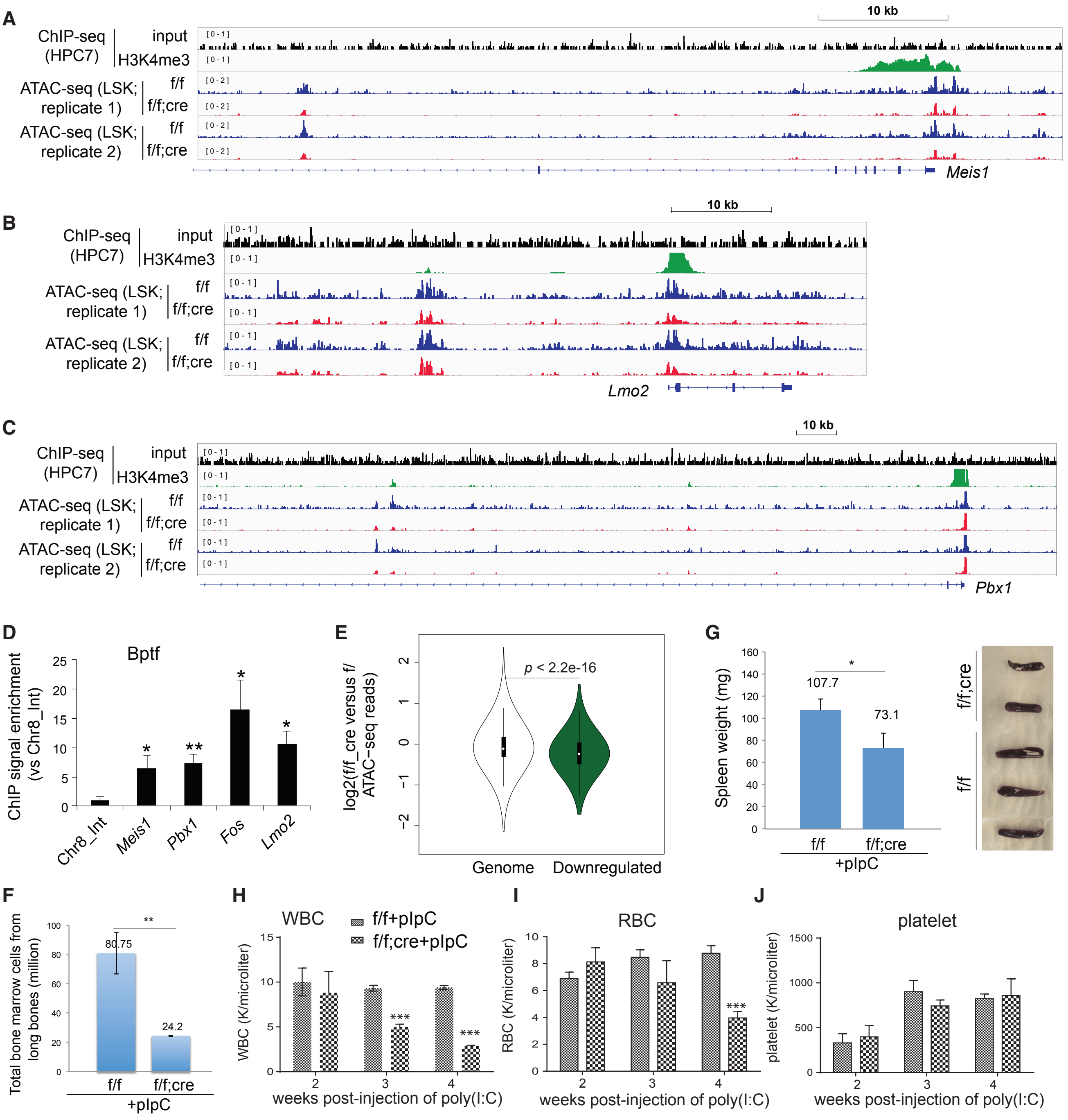

Figure 4. BPTF Potentiates Chromatin Accessibility at HSC "Stemness" Genes

(A–C) ChIP-seq profiles of H3K4me3 and input at the indicated genes in HPC-7 cells, and their ATAC-seq profiles in *Bptf*$^{f/f}$ versus *Bptf*^{cKO} LSK cells 7 days after cre induction. For cross-sample comparison, the scales of profiles are normalized with total sequencing read counts (see also Figure S3A).

(D) BPTF ChIP at the indicated gene promoter in HPC-7 cells. Fold of enrichment in signals, shown as mean ± SD (n = 3 biological replicates), was normalized to input and to a control locus (Chr8_Int). $*p < 0.05$; $**p < 0.01$.

(E) Comparison of ATAC-seq data in *Bptf*^{cKO} versus *Bptf*$^{f/f}$ LSK cells shows a significant reduction of ATAC-seq signals at the promoters of genes showing downregulation due to BPTF loss, relative to genome background. Plotted at y axis are $\log_2$-transformed ratios of promoter-associated ATAC-seq reads between two samples, either at all genes (left) or at the top 500 downregulated genes in *Bptf*^{cKO} LSK cells (right), relative to *Bptf*$^{f/f}$ control (see also Figures S3B and S3C).

(legend continued on next page)

cre induction (Figure S3B). Upon *Bptf* ablation, we did not see a dramatic change in global ATAC-seq signals (Figure S3C) but observed significantly reduced DNA accessibility at the promoters of downregulated genes (Figure 4E) such as *Meis1*, *Pbx1*, *Fos*, and *Lmo2* (Figures 4A–4C and S3A). We also observed decreased ATAC-seq signals at putative distal or intragenic enhancers of these TF genes (Figures 4A–4C and S3A). Together, these genomic data support crucial roles of BPTF in potentiating DNA accessibility and appropriate expression of key HSC TF genes. Further work is needed to firmly define genomic binding of BPTF in HSPCs.

Hematopoietic-Specific Loss of BPTF Leads to Bone Marrow Failure, Anemia, and Leukopenia

Given the impaired function of $Bptf^{cKO}$ HSCs, we predicted that lineage-committed populations in $Bptf^{cKO}$ mice would be affected. Four weeks after *Bptf* deletion, the bones from $Bptf^{cKO}$ mice appeared pale and showed significant decrease in the total BM cell number when compared with control (Figure 4F). Moreover, we observed that the $Bptf^{cKO}$ mice possessed smaller spleens relative to control, which suggests a defect in splenic B cell development (Figure 4G). FACS of total splenic cells confirmed our observation, with a significant reduction in the $B220^+$ cells (Figure S4A). Furthermore, complete blood counts revealed anemia, leukopenia, and granulocytopenia in $Bptf^{cKO}$ mice, phenotypes that arise from the dysfunctional repopulation capacity of HSCs (Figures 4H–4J and S4B–S4D). Thus, we show *Bptf* to be essential for normal hematopoiesis.

DISCUSSION

BPTF Plays an Essential Role in the Maintenance and Functionality of HSCs

How adult stem cells sustain themselves remains as an intriguing question. Using knockout and reconstitution systems, we showed an essential requirement of BPTF for maintaining the HSPC populations and their repopulating capacity. Mechanistically, our transcriptome profiling revealed a previously unappreciated, BPTF-dependent gene-activation program, which includes a set of master TFs known to be vital for HSC self-renewal (*Meis1*, *Pbx1*, *Mn1*, and *Lmo2*), the AP1 complex, and the MLL/KMT2A signature genes. BPTF also sustains an open chromatin state at target "stemness" genes. Thus, BPTF acts as a safeguard of adult hematopoiesis, ensuring HSCs' reconstitution function. In support, BPTF loss caused BM failure phenotypes that are reminiscent of what was observed for *KMT2A*-null HSCs (Artinger et al., 2013; Jude et al., 2007). BPTF appears to be more crucial for blood formation under stressed conditions (e.g., reconstitution in irradiated mice) than in the steady state, a phenomenon also described in a conditional *KMT2A*-null model (McMahon et al., 2007). However, depth study is required to dissect the potentially overlapping and distinctive roles for BPTF and KMT2 in HSC self-renewal and blood formation.

BPTF Controls Vital Gene-Expression Programs to Sustain Homeostasis of Multiple Cell Lineages

In related research, BPTF acts as a crucial regulator of mammary gland and epidermal stem cells (Frey et al., 2017; Mulder et al., 2012), melanocytes (Koludrovic et al., 2015), and T cells (Landry et al., 2011; Wu et al., 2016). Here, an intriguing question is how the general chromatin regulator BPTF controls a defined yet distinct gene-expression program among different cell lineages. Presumably these cells differ in patterns of histone modifications, which can stabilize BPTF binding to genes essential for lineage definition. Also, BPTF/NURF interacts with DNA-binding factors such as CTCF (Qiu et al., 2015) and c-MYC (Richart et al., 2016a). A multivalent interaction of NURF to histone modifications, TFs, and other recruiting factors can act in concert to dictate distinct genomic targeting of BPTF/NURF.

The Essential Function of BPTF in Normal Tissue Raises a Concern on Targeting It in Cancer Therapy

Recently, the oncogenic role of BPTF was reported in melanoma (Dar et al., 2015, 2016), pancreatic tumors, and Burkitt's lymphoma (Richart et al., 2016b), where BPTF was shown to promote the gene program related to tumor cell growth or survival such as c-MYC and BCL2. Bptf carries an H3K4me3-binding PHD and an acetyl-histone-binding bromodomain. Both motifs including PHD associate with human disease (Baker et al., 2008; Gough et al., 2014; Wang et al., 2009) and can be potentially druggable (Arrowsmith et al., 2012). BPTF was proposed as a drug target for cancer therapy (Richart et al., 2016a). However, increasing evidence now shows a vital requirement of BPTF for normal homeostasis of a range of tissues. Such broad homeostatic function for Bptf requires additional studies to address toxicity associated with targeting this protein in cancer.

(F and G) Total cell numbers in the femur (F) and the size of spleen (G) in $Bptf^{f/f}$ (n = 3) versus $Bptf^{cKO}$ (n = 4) mice 4 weeks after cre induction. *p < 0.05; **p < 0.01.
(H–J) Complete blood counts of peripheral blood collected from $Bptf^{cKO}$ mice (n = 4) and $Bptf^{f/f}$ littermates (n = 4): WBC, white blood cells (H); RBC, red blood cells (I); and platelets (J) (see also Figure S4). ***p < 0.001.

EXPERIMENTAL PROCEDURES

Details of additional procedures such as BMT, FACS, sorting, ATAC-seq, and ChIP are provided in Supplemental Experimental Procedures.

Knockout Mice

CD45.2[+] B/6 mice carrying the *Bptf*[f/f] allele (stock #009367) or *Mx1*-cre were purchased from the Jackson Laboratory and crossed to produce *Bptf*[f/f];*Mx1*-cre mice and control littermates. To induce *Bptf* knockout in the BM, we injected 2- to 3-month-old mice with pIpC (Sigma) three times every other day. UNC-Chapel Hill Institutional Animal Care and Use Committee approved all animal experiments.

RNA-Seq

RNA was extracted from sorted LSK cells with the picoRNA Kit (Applied Biosystems) and the RNA-seq library was prepared with Illumina kits according to the manufacturer's protocol, followed by deep sequencing.

ACCESSION NUMBERS

The genomics data are deposited to the NCBI GEO repository under GEO: GSE108441.

SUPPLEMENTAL INFORMATION

Supplemental Information includes Supplemental Experimental Procedures, four figures, and two tables and can be found with this article online at https://doi.org/10.1016/j.stemcr.2018.01.020.

AUTHOR CONTRIBUTIONS

G.G.W. designed the project. B.X., L.C., J.M.B., X.L., D.F.A., R.L., S.R., and G.G.W. performed experiments and interpreted data. J.S.P., D.Z., and D.C. analyzed genomic data. G.G.W. wrote the manuscript, and D.F.A. and B.X. critically read the paper.

ACKNOWLEDGMENTS

We thank L. Carlsson for providing HPC-7 cells and UNC Cores of FACS, Animal Studies, Sequencing and Bioinformatics for their support. This work is funded by NCI grants R01CA211336, R01CA218600, and R01CA215284 (to G.G.W). UNC Cores are supported partly by UNC Cancer Center Core support grant P30-CA016086. G.G.W. is a Junior Faculty Scholar of American Society of Hematology and a Kimmel Scholar and a Research Scholar of American Cancer Society and received funding support from Gabrielle's Angel Foundation and Concern Foundation.

Received: October 22, 2017
Revised: January 19, 2018
Accepted: January 19, 2018
Published: February 15, 2018

REFERENCES

Arrowsmith, C.H., Bountra, C., Fish, P.V., Lee, K., and Schapira, M. (2012). Epigenetic protein families: a new frontier for drug discovery. Nat. Rev. Drug Discov. *11*, 384–400.

Artinger, E.L., Mishra, B.P., Zaffuto, K.M., Li, B.E., Chung, E.K., Moore, A.W., Chen, Y., Cheng, C., and Ernst, P. (2013). An MLL-dependent network sustains hematopoiesis. Proc. Natl. Acad. Sci. USA *110*, 12000–12005.

Baker, L.A., Allis, C.D., and Wang, G.G. (2008). PHD fingers in human diseases: disorders arising from misinterpreting epigenetic marks. Mutat. Res. *647*, 3–12.

Bock, C., Beerman, I., Lien, W.H., Smith, Z.D., Gu, H., Boyle, P., Gnirke, A., Fuchs, E., Rossi, D.J., and Meissner, A. (2012). DNA methylation dynamics during in vivo differentiation of blood and skin stem cells. Mol. Cell *47*, 633–647.

Chambers, S.M., Boles, N.C., Lin, K.Y., Tierney, M.P., Bowman, T.V., Bradfute, S.B., Chen, A.J., Merchant, A.A., Sirin, O., Weksberg, D.C., et al. (2007). Hematopoietic fingerprints: an expression database of stem cells and their progeny. Cell Stem Cell *1*, 578–591.

Chi, P., Allis, C.D., and Wang, G.G. (2010). Covalent histone modifications—miswritten, misinterpreted and mis-erased in human cancers. Nat. Rev. Cancer *10*, 457–469.

Dar, A.A., Majid, S., Bezrookove, V., Phan, B., Ursu, S., Nosrati, M., De Semir, D., Sagebiel, R.W., Miller, J.R., 3rd, Debs, R., et al. (2016). BPTF transduces MITF-driven prosurvival signals in melanoma cells. Proc. Natl. Acad. Sci. USA *113*, 6254–6258.

Dar, A.A., Nosrati, M., Bezrookove, V., de Semir, D., Majid, S., Thummala, S., Sun, V., Tong, S., Leong, S.P., Minor, D., et al. (2015). The role of BPTF in melanoma progression and in response to BRAF-targeted therapy. J. Natl. Cancer Inst. *107*.

Dou, Y., Milne, T.A., Tackett, A.J., Smith, E.R., Fukuda, A., Wysocka, J., Allis, C.D., Chait, B.T., Hess, J.L., and Roeder, R.G. (2005). Physical association and coordinate function of the H3 K4 methyltransferase MLL1 and the H4 K16 acetyltransferase MOF. Cell *121*, 873–885.

Frey, W.D., Chaudhry, A., Slepicka, P.F., Ouellette, A.M., Kirberger, S.E., Pomerantz, W.C.K., Hannon, G.J., and Dos Santos, C.O. (2017). BPTF maintains chromatin accessibility and the self-renewal capacity of mammary gland stem cells. Stem Cell Reports *9*, 23–31.

Gough, S.M., Lee, F., Yang, F., Walker, R.L., Zhu, Y.J., Pineda, M., Onozawa, M., Chung, Y.J., Bilke, S., Wagner, E.K., et al. (2014). NUP98-PHF23 is a chromatin-modifying oncoprotein that causes a wide array of leukemias sensitive to inhibition of PHD histone reader function. Cancer Discov. *4*, 564–577.

Heuser, M., Yun, H., Berg, T., Yung, E., Argiropoulos, B., Kuchenbauer, F., Park, G., Hamwi, I., Palmqvist, L., Lai, C.K., et al. (2011). Cell of origin in AML: susceptibility to MN1-induced transformation is regulated by the MEIS1/AbdB-like HOX protein complex. Cancer Cell *20*, 39–52.

Jude, C.D., Climer, L., Xu, D., Artinger, E., Fisher, J.K., and Ernst, P. (2007). Unique and independent roles for MLL in adult hematopoietic stem cells and progenitors. Cell Stem Cell *1*, 324–337.

Kadoch, C., and Crabtree, G.R. (2015). Mammalian SWI/SNF chromatin remodeling complexes and cancer: mechanistic insights gained from human genomics. Sci. Adv. *1*, e1500447.

Koludrovic, D., Laurette, P., Strub, T., Keime, C., Le Coz, M., Coassolo, S., Mengus, G., Larue, L., and Davidson, I. (2015). Chromatin-remodelling complex NURF is essential for differentiation of adult melanocyte stem cells. PLoS Genet. *11*, e1005555.

Krivtsov, A.V., Twomey, D., Feng, Z., Stubbs, M.C., Wang, Y., Faber, J., Levine, J.E., Wang, J., Hahn, W.C., Gilliland, D.G., et al. (2006). Transformation from committed progenitor to leukaemia stem cell initiated by MLL-AF9. Nature *442*, 818–822.

Landry, J., Sharov, A.A., Piao, Y., Sharova, L.V., Xiao, H., Southon, E., Matta, J., Tessarollo, L., Zhang, Y.E., Ko, M.S., et al. (2008). Essential role of chromatin remodeling protein Bptf in early mouse embryos and embryonic stem cells. PLoS Genet. *4*, e1000241.

Landry, J.W., Banerjee, S., Taylor, B., Aplan, P.D., Singer, A., and Wu, C. (2011). Chromatin remodeling complex NURF regulates thymocyte maturation. Genes Dev. *25*, 275–286.

Large, E.E., Xu, W., Zhao, Y., Brady, S.C., Long, L., Butcher, R.A., Andersen, E.C., and McGrath, P.T. (2016). Selection on a subunit of the NURF chromatin remodeler modifies life history traits in a domesticated strain of *Caenorhabditis elegans*. PLoS Genet. *12*, e1006219.

Lim, D.A., Huang, Y.C., Swigut, T., Mirick, A.L., Garcia-Verdugo, J.M., Wysocka, J., Ernst, P., and Alvarez-Buylla, A. (2009). Chromatin remodelling factor Mll1 is essential for neurogenesis from postnatal neural stem cells. Nature *458*, 529–533.

McMahon, K.A., Hiew, S.Y., Hadjur, S., Veiga-Fernandes, H., Menzel, U., Price, A.J., Kioussis, D., Williams, O., and Brady, H.J. (2007). Mll has a critical role in fetal and adult hematopoietic stem cell self-renewal. Cell Stem Cell *1*, 338–345.

Mulder, K.W., Wang, X., Escriu, C., Ito, Y., Schwarz, R.F., Gillis, J., Sirokmany, G., Donati, G., Uribe-Lewis, S., Pavlidis, P., et al. (2012). Diverse epigenetic strategies interact to control epidermal differentiation. Nat. Cell Biol. *14*, 753–763.

Qiu, Z., Song, C., Malakouti, N., Murray, D., Hariz, A., Zimmerman, M., Gygax, D., Alhazmi, A., and Landry, J.W. (2015). Functional interactions between NURF and Ctcf regulate gene expression. Mol. Cell. Biol. *35*, 224–237.

Richart, L., Carrillo-de Santa Pau, E., Río-Machín, A., de Andrés, M.P., Cigudosa, J.C., Lobo, V.J., and Real, F.X. (2016a). BPTF is required for c-MYC transcriptional activity and in vivo tumorigenesis. Nat. Commun. *7*, 10153.

Richart, L., Real, F.X., and Sanchez-Arevalo Lobo, V.J. (2016b). c-MYC partners with BPTF in human cancer. Mol. Cell. Oncol. *3*, e1152346.

Ruthenburg, A.J., Li, H., Milne, T.A., Dewell, S., McGinty, R.K., Yuen, M., Ueberheide, B., Dou, Y., Muir, T.W., Patel, D.J., et al. (2011). Recognition of a mononucleosomal histone modification pattern by BPTF via multivalent interactions. Cell *145*, 692–706.

Schwanbeck, R., Xiao, H., and Wu, C. (2004). Spatial contacts and nucleosome step movements induced by the NURF chromatin remodeling complex. J. Biol. Chem. *279*, 39933–39941.

Seita, J., Sahoo, D., Rossi, D.J., Bhattacharya, D., Serwold, T., Inlay, M.A., Ehrlich, L.I., Fathman, J.W., Dill, D.L., and Weissman, I.L. (2012). Gene Expression Commons: an open platform for absolute gene expression profiling. PLoS One *7*, e40321.

Stankiewicz, P., Khan, T.N., Szafranski, P., Slattery, L., Streff, H., Vetrini, F., Bernstein, J.A., Brown, C.W., Rosenfeld, J.A., Rednam, S., et al. (2017). Haploinsufficiency of the chromatin remodeler BPTF causes syndromic developmental and speech delay, postnatal microcephaly, and dysmorphic features. Am. J. Hum. Genet. *101*, 503–515.

Wang, G.G., Allis, C.D., and Chi, P. (2007). Chromatin remodeling and cancer, Part II: ATP-dependent chromatin remodeling. Trends Mol. Med. *13*, 373–380.

Wang, G.G., Pasillas, M.P., and Kamps, M.P. (2005). Meis1 programs transcription of FLT3 and cancer stem cell character, using a mechanism that requires interaction with Pbx and a novel function of the Meis1 C-terminus. Blood *106*, 254–264.

Wang, G.G., Song, J., Wang, Z., Dormann, H.L., Casadio, F., Li, H., Luo, J.L., Patel, D.J., and Allis, C.D. (2009). Haematopoietic malignancies caused by dysregulation of a chromatin-binding PHD finger. Nature *459*, 847–851.

Wilson, N.K., Foster, S.D., Wang, X., Knezevic, K., Schutte, J., Kaimakis, P., Chilarska, P.M., Kinston, S., Ouwehand, W.H., Dzierzak, E., et al. (2010). Combinatorial transcriptional control in blood stem/progenitor cells: genome-wide analysis of ten major transcriptional regulators. Cell Stem Cell *7*, 532–544.

Wu, B., Wang, Y., Wang, C., Wang, G.G., Wu, J., and Wan, Y.Y. (2016). BPTF is essential for T cell homeostasis and function. J. Immunol. *197*, 4325–4333.

Wysocka, J., Swigut, T., Xiao, H., Milne, T.A., Kwon, S.Y., Landry, J., Kauer, M., Tackett, A.J., Chait, B.T., Badenhorst, P., et al. (2006). A PHD finger of NURF couples histone H3 lysine 4 trimethylation with chromatin remodelling. Nature *442*, 86–90.

Stem Cell Reports, Volume *10*

Supplemental Information

The Chromatin Remodeler BPTF Activates a Stemness Gene-Expression Program Essential for the Maintenance of Adult Hematopoietic Stem Cells

Bowen Xu, Ling Cai, Jason M. Butler, Dongliang Chen, Xiongdong Lu, David F. Allison, Rui Lu, Shahin Rafii, Joel S. Parker, Deyou Zheng, and Gang Greg Wang

Supplemental Information

The chromatin remodeler BPTF activates a stemness gene-expression program essential for the maintenance of adult hematopoietic stem cells

Bowen Xu et al.

Inventory of Supplemental Information

Supplemental Figures S1-S4 and Legends

Figure S1 related to main Fig 1 and 2

Figure S2 related to main Fig 3

Figure S3-4 related to main Fig 4

Table S1 related to main Fig 3

Table S2 related to main Fig 1, 3 and 4

Supplemental Experimental Procedures

Reference

Figure S1

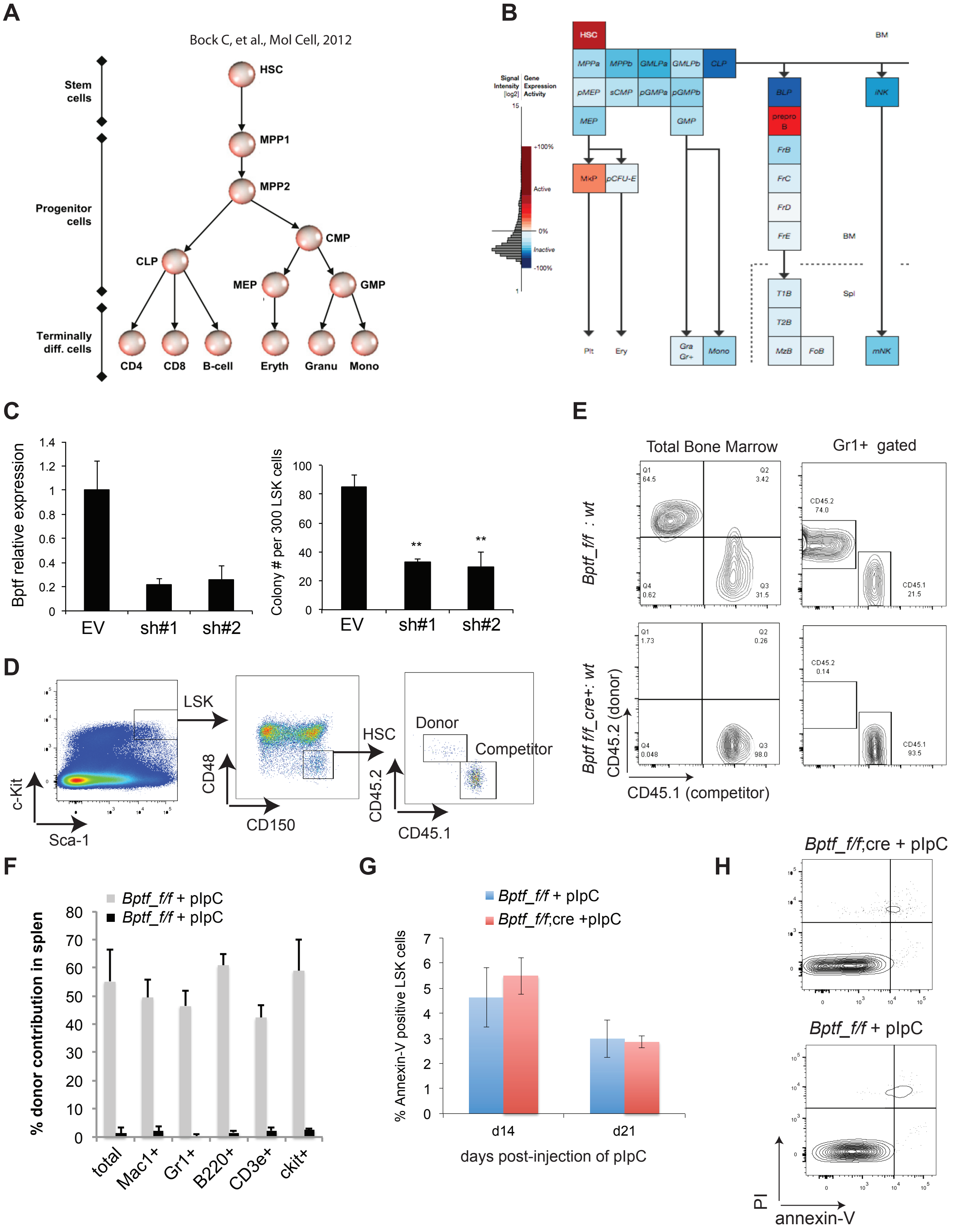
A
Bock C, et al., Mol Cell, 2012
Stem cells
Progenitor cells
Terminally diff. cells
HSC
MPP1
MPP2
CLP
CMP
MEP
GMP
CD4
CD8
B-cell
Eryth
Granu
Mono
B
HSC
MPPa
MPPb
GMLPa
GMLPb
CLP
pMEP
sCMP
pGMPa
pGMPb
MEP
GMP
MkP
pCFU-E
BLP
prepro B
FrB
FrC
FrD
FrE
T1B
T2B
MzB
FoB
iNK
mNK
Plt
Ery
Gra Gr+
Mono
BM
Spl
C
Bptf relative expression
EV
sh#1
sh#2
Colony # per 300 LSK cells
D
LSK
HSC
Donor
Competitor
c-Kit
Sca-1
CD48
CD150
CD45.2
CD45.1
E
Total Bone Marrow
Gr1+ gated
Bptf_f/f : wt
Bptf f/f_cre+: wt
CD45.2 (donor)
CD45.1 (competitor)
F
Bptf_f/f + pIpC
Bptf_f/f + pIpC
% donor contribution in splen
total
Mac1+
Gr1+
B220+
CD3e+
ckit+
G
Bptf_f/f + pIpC
Bptf_f/f;cre +pIpC
% Annexin-V positive LSK cells
d14
d21
days post-injection of pIpC
H
Bptf_f/f;cre + pIpC
Bptf_f/f + pIpC
PI
annexin-V

Figure S1. Maintenance of the HSPC compartments and their reconstitution capacities requires *Bptf* expression.

(A) Hematopoietic cell subtypes shown in main Figure 1A, with their expression dataset obtained from a published study (Bock et al., 2012). **(B)** *Bptf* expression (microarray probe: 1427311_at) during hematopoietic development according to the "Gene Expression Commons" dataset (Seita et al., 2012). **(C)** Effect of *Bptf* knockdown by either of the two validated shRNA (left panel) on colony formation (right). Plotted in the right panel were mean ± SD (n= 3 biological replicates) of CFU colonies derived from 300 of the LSK cells stably transduced with empty vector control (EV) or shRNA. Statistical analysis was determined by two-tailed Student's t-test: *, $p<0.05$; **, $p<0.01$. **(D)** A representative FACS scheme detecting either the wild-type competitor-derived CD45.1+ cells or the donor-derived CD45.2+ cells. Shown are $Bptf^{f/f}$;*Mx1*-cre+ ($Bptf^{cKO}$) donor-derived cells analyzed five weeks after cre induction among the LSK and LT-HSC (LSK/CD150+/CD48-) subpopulations in the BM. **(E)** Typical FACS plots of the donor-derived CD45.2+ cells, either from $Bptf^{f/f}$ or $Bptf^{cKO}$ mice, in the indicated BM cells eight weeks after cre induction. **(F)** Summary of average percentages of the donor-derived CD45.2+ cells, either from the control $Bptf^{f/f}$ (n=4) or $Bptf^{cKO}$ (n=4) mice, among the indicated spleen cells eight weeks after cre induction. **(G-H)** Summary (panel **G;** n=3 mice) and FACS plot (panel **H**) of the annexin-V+ apoptotic cells among the LSK populations at the indicated time point after cre induction.

Figure S2

A

Summary of LSK cell RNA-seq read tags

Sample	Replicate	Total # of reads
f/f+pIpC	1	36,604,132
	2	20,226,077
f/f;cre+pIpC	1	44,658,576
	2	20,573,687

C

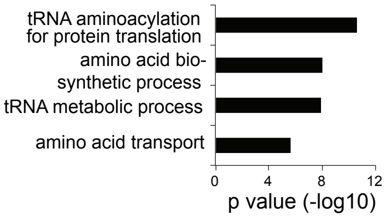

B

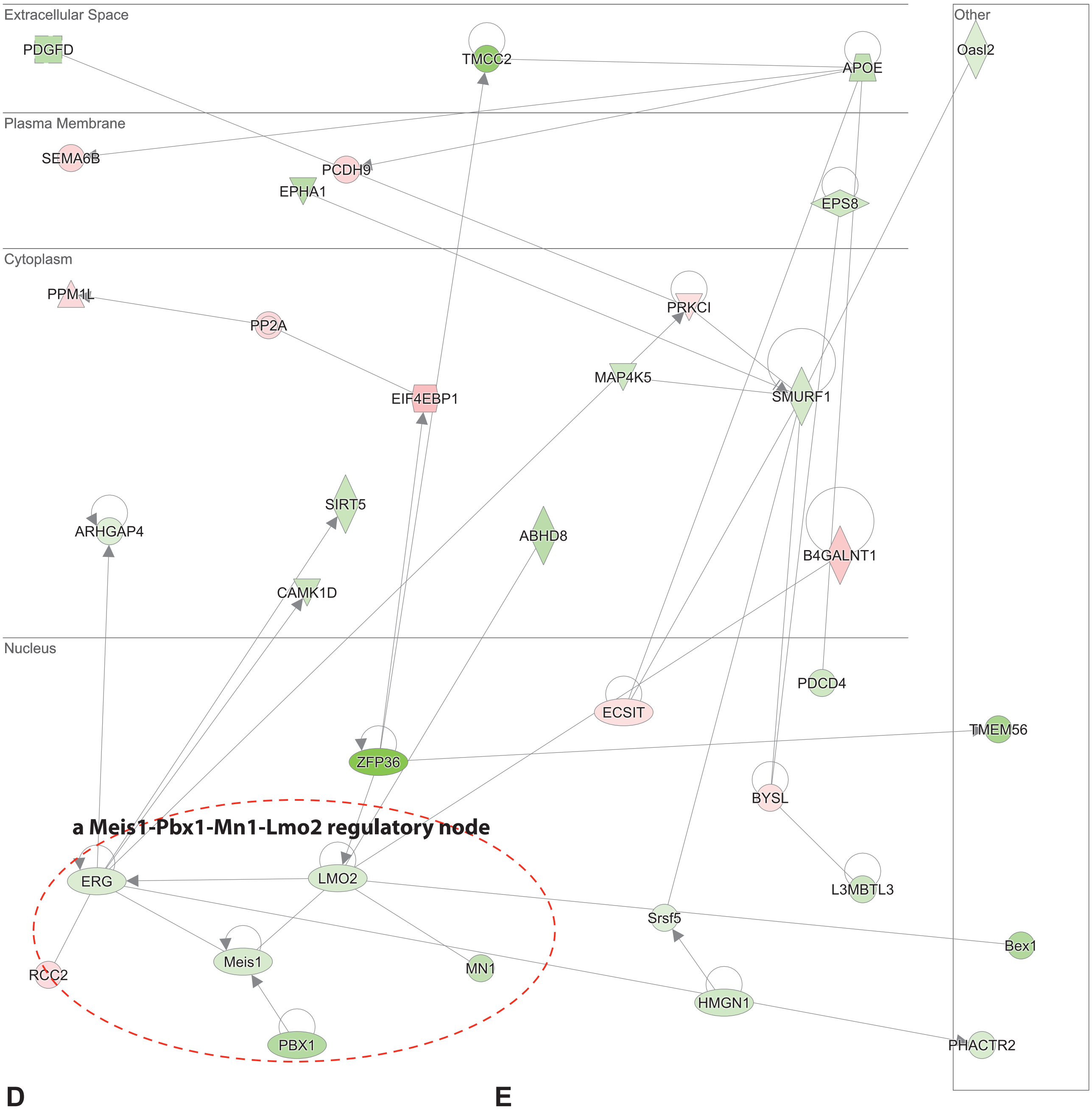

D

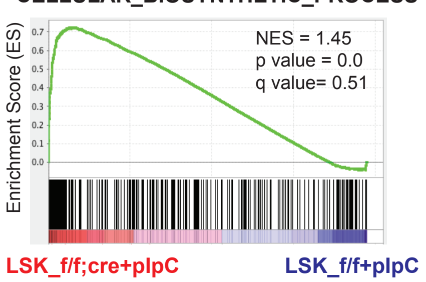

E

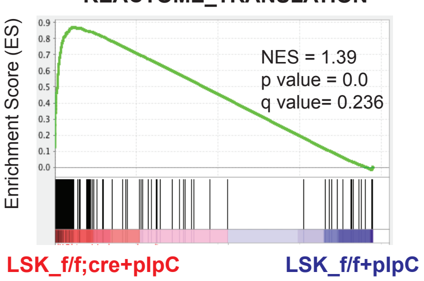

Figure S2. RNA-seq profiling identifies an unappreciated, BPTF-dependent transcriptional activation program that includes a crucial 'stemess' regulatory node consisting of several HSC master regulators.

(A) Summary of read counts from RNA-sequencing (RNA-seq) profiles of the control (*Bptf*$^{f/f}$) and *Bptf*^{cKO} (*Bptf*$^{f/f}$*;Mx1*-cre) LSK cells that were sorted from 5 mice (in two biological replicates) on day 10 after cre induction.

(B) Analysis of the deregulated genes with the Ingenuity Pathway Analysis (IPA) tool identifies the significant perturbation (p<10E-47) of the shown molecular pathway that is related to cancer and development. Green and red show the down- and up-regulated genes, respectively, in the *Bptf*^{cKO} LSK cells relative to *Bptf*$^{f/f}$ controls, with genes categorized according to their subcellular localizations.

(C) GO analysis reveals the indicated gene pathways among the transcripts found up-regulated in the *Bptf*^{cKO} LSK cells relative to *Bptf*$^{f/f}$ controls.

(D-E) GSEA reveals enrichment of the indicated gene signature in the *Bptf*$^{f/f}$ versus *Bptf*^{cKO} LSK cells isolated from mice after cre induction.

Figure S3

A

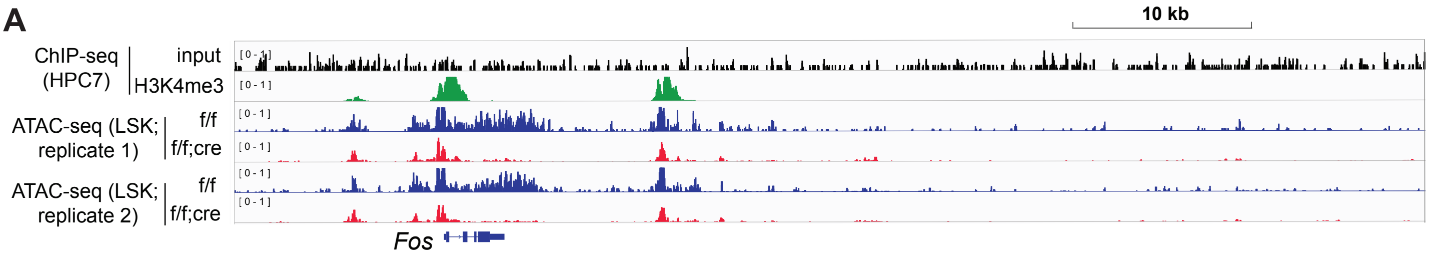

B

Summary of LSK cell ATAC-seq read tags

	Cell types	File ID	Total # of Reads
replicate #1	LSK control (Bptf_f/f)	ATAC-BPWT_150225	10,024,904
	LSK Bptf_cKO	ATAC-BPKO_150225	21,874,128
replicate #2	LSK control (Bptf_f/f)	ATAC-BPWT_150319	16,582,933
	LSK Bptf_cKO	ATAC-BPKO_150319	37,729,978
replicate #3	LSK control (Bptf_f/f)	ATAC-BPWT_150130	8,441,275
	LSK Bptf_cKO	ATAC-BPKO_150130	18,397,932

C

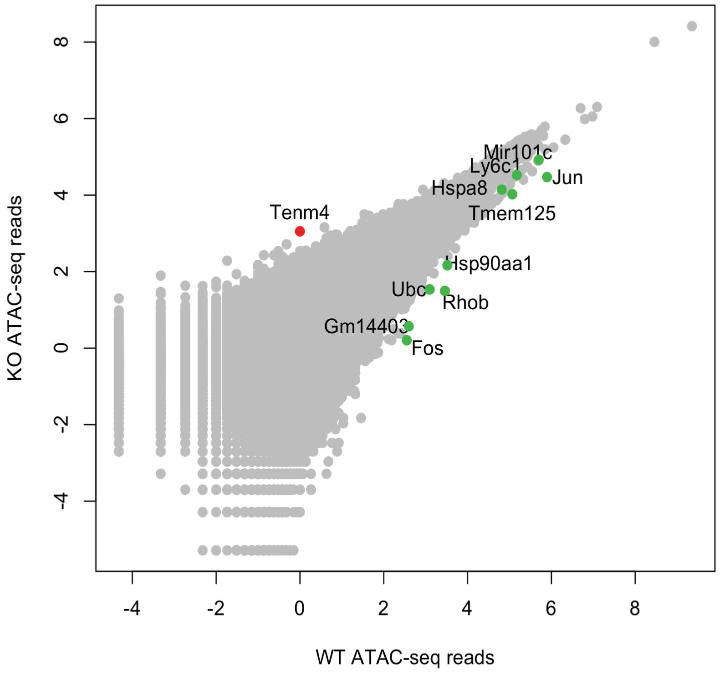

Figure S3. BPTF potentiates chromatin accessibility of key HSC 'stemess' TF genes.

(A) ChIP-seq profiles showing H3K4me3 and the chromatin inputs at the indicated gene in HPC-7 cells, as well as the ATAC-seq profiles among the control versus *Bptf*^{cKO} LSK cells that were sorted on day 7 after cre induction. For cross-sample comparison, the scales of profiles are normalized with total sequencing read counts.

(B) Summary of read counts from ATAC-sequencing (ATAC-seq) profiles of the control (*Bptf*$^{f/f}$) and *Bptf*^{cKO} (*Bptf*$^{f/f}$;*Mx1*-cre) LSK cells on day 7 after cre induction.

(C) Comparison of global DNA accessibility among the *Bptf*^{cKO} (y-axis) versus *Bptf*$^{f/f}$ (x-axis) LSK cells on day 7 after cre induction. Plotted at x- and y-axis are ATAC-seq read counts for each called individual peak in log2 transformation of per million sequencing depth.

Figure S4

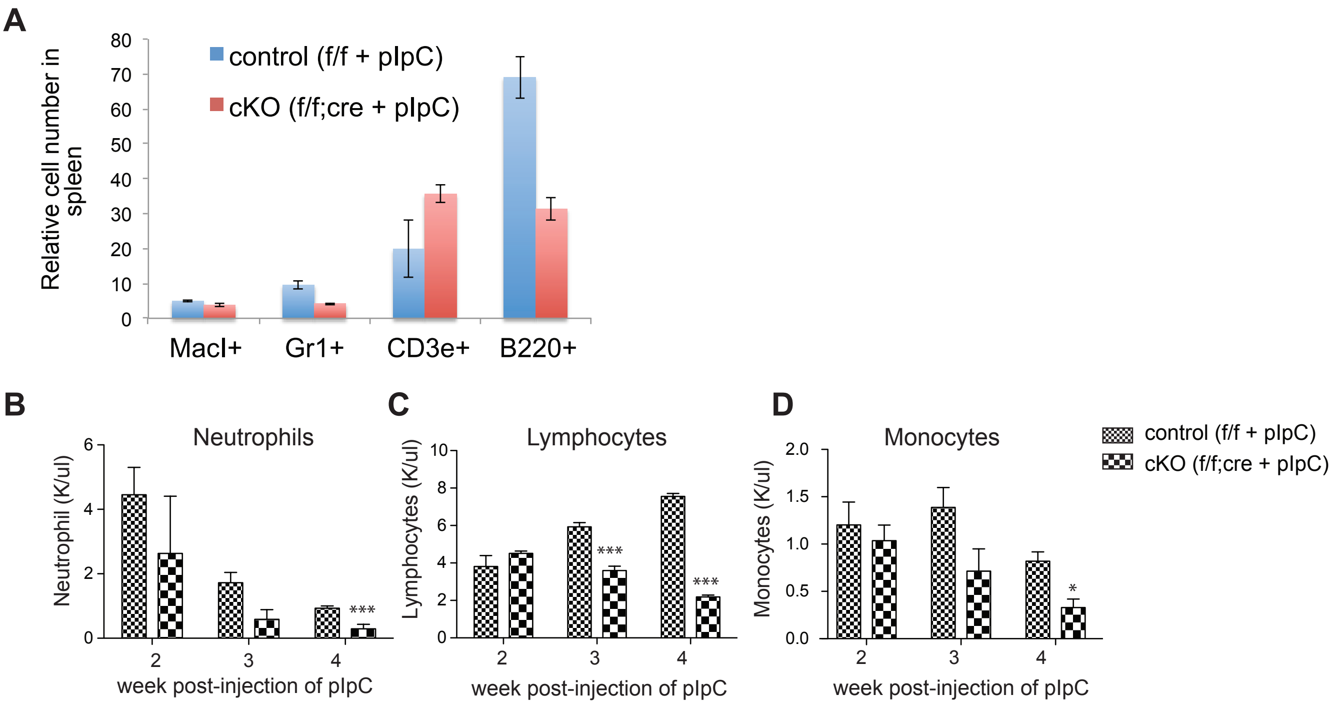
A
Relative cell number in spleen
80
70
60
50
40
30
20
10
0
control (f/f + pIpC)
cKO (f/f;cre + pIpC)
MacI+
Gr1+
CD3e+
B220+
B
Neutrophils
Neutrophil (K/ul)
6
4
2
0
***
2
3
4
week post-injection of pIpC
C
Lymphocytes
Lymphocytes (K/ul)
10
8
6
4
2
0
***
***
2
3
4
week post-injection of pIpC
D
Monocytes
Monocytes (K/ul)
2.0
1.5
1.0
0.5
0.0
*
2
3
4
week post-injection of pIpC
control (f/f + pIpC)
cKO (f/f;cre + pIpC)

Figure S4. *Bptf* loss in the hematopoietic compartment causes severe defects in the reconstitution capacities of HSPCs, anemia and leukopenia.

(A) Summary of averaged cell numbers of the indicated splenic cell populations isolated from the control (*Bptf*$^{f/f}$, n=3) and *Bptf*^{cKO} (n=4) mice, as analyzed by FACS.

(B-D) Complete blood cell counts of peripheral blood collected from the *Bptf*^{cKO} mice and control (*Bptf*$^{f/f}$) littermates (n=4 mice) at the indicated time after cre induction.

Table S2. Primer information. Related to Figure 1, 3 and 4.

	Gene	Forward	Reverse	Note
**RT-qPCR**				
	Bptf	agcagcttcaggagccatag<br>cgttcgacacgaacctattg	aggggtgggatgtctttttc<br>gctaactggacctttgtgctg	target exon-2 to check deletion efficiency of BPTF cKO
	Fos (c-Fos)	TTTCAACGCCGACTACGAGG	GCGCAAAAGTCCTGTGTGTT	
	Jun	TGGGCACATCACCACTACAC	TCTGGCTATGCAGTTCAGCC	
	Pbx1	GCGCCGGGAGCCCATTTCTGC	GGTCCCTCCGGCCCCATCCTG	
	Meis1	AAGGTGATGGCTTGGACAAC	TGTGCCAACTGCTTTTTCTG	
	Lmo2	CTGGACCCGTCTGAGGAAC	GTCACAGCTGAGGCAATCCT	
	Hoxa5	GCAAGCTGCACATTAGTCAC	GCATGAGCTATTTCGATCCT	
	Hoxa9	ACAATGCCGAGAATGAGAGC	CAGCGTCTGGTGTTTTGTGT	
	Cd34	CTGGAATCCGAGAAGTGAGG	GCCTCCTCCTTTTCACACAG	
	cKit	ATGAACAGGACCTCGGCTAAC	ACTGGCATCAGAGTTGGACAC	
	Hprt1	TCCTCCTCAGACCGCTTTT	CCTGGTTCATCATCGCTAATC	internal control
	beta-Actin	ACCAACTGGGACGACATGGA	GGTCTCAAACATGATCTGGGTCAT	internal control
	Gapdh	TGCACCACCAACTGCTTAGC	GGCATGGACTGTGGTCATGAG	internal control
**ChIP-qPCR**				
	Meis1	gcagttgcaaagagggagag	gcccgctttccttgaatc	near TSS
	Pbx1	CTCCTCCTCCTCCTCTCAGG	CTCTTTCCTCCTCCGTGTCTC	near TSS
	Fos	AATCCTACACGCGGAAGGTC	CCCCGTCTTGGCATACATCT	near TSS
	Chr8_int	AAGGGGCCTCTGCTTAAAAA	AGAGCTCCATGGCAGGTAGA	an intergenic region of chr 8 as negative control

Supplemental Methods

Mouse. Genotyping information for *Bptf*$^{f/f}$ and *Mx1*-cre alleles is available from the Jackson Laboratory. CD45.1+ B/6 congenic mice (B6-LY5.2/Cr) were purchased from NCI Frederick Mouse Repository. UNC-Chapel Hill Institutional Animal Care and Use Committee approved all animal experiments.

Bone marrow transplantation (BMT) and in vivo competitive reconstitution assay. Total BM cells isolated from femur and tibia of donor (CD45.2+) and competitor (CD45.1+) b/6 mice were treated with the ACK lysis buffer (0.15 M NH4Cl, 10 mM KHCO3, 0.1 mM EDTA [pH 7.2]) to remove red blood cells. An equal amount mixture of unfractionated donor and competitor BM cells was transplanted via tail veil injection into the lethally irradiated, congenic CD45.1+ b/6 recipient mice (irradiated with a single dose of 1,000 rads; carried out by the Animal Studies Core affiliated to UNC Cancer Center). Stable engraftment of donor (CD45.2+) and competitor (CD45.1+) BM cells was monitored by FACS analysis of the peripheral blood (PB) cells. Briefly, about 20ul of PB was collected from tail veil nick, subject to ACK lysis, and stained with antibodies of CD45.2-APC (ebioscience, cat# 17-0454) and CD45.1-FITC (ebioscience, cat# 11-0453). To induce gene deletion, the experimental BMT cohort (i.e. engrafted with *Bptf*$^{f/f}$*;Mx1-cre* donor BM cells) and control cohorts (i.e. engrafted with *Bptf*$^{f/f}$ alone or the heterozygous *Bptf*$^{f/w}$*;Mx1*-cre donor BM cells) were subject to poly(I:C) injection as described before (Li et al., 2011). The percentages of donor(CD45.2+)-derived and competitor(CD45.1+)-derived cells were analyzed with FACS in the PB or BM. For FACS of LSK cells, about 10 million total BM cells were harvested and stained with FITC-labeled antibody cocktail for lineage markers (CD11b-FITC [eBioscience 11-0112-82], Gr1-FITC [eBioscience 11-5931-82], CD3e-FITC [eBioscience 11-0031-82],

Ter119-FITC [eBioscience 11-5921-82], B220-FITC [eBioscience 11-0452-82], CD8a-FITC [eBioscience 11-0081-82], CD5-FITC [eBioscience 11-0051-81], CD2-FITC [eBioscience 11-0021-81], CD4-FITC [eBioscience 11-0042-82]), cKIT-APC (BD Biosciences 561074), SCA1-PE-Cy7 (BD Biosciences 558162), CD150-PE (eBioscience 12-1502), CD48-PerCP-Cy5.5 (BioLegend 103422), CD45.2-Alexa Fluor 700 (eBioscience 56-0454-82), and CD45.1-APC-eFluor 780 (eBioscience 47-0453-82). Samples were analyzed with a LSRII machine (BD Biosciences; UNC flow core).

Flow cytometry (FACS) analysis and cell sorting. Total BM cells were isolated from the femur and tibia of mice. FACS-based analysis of lineage-/SCA-1+/cKIT+ (LSK) cells and long-term hematopoietic stem cells (LT-HSC) were carried out using LSRII (BD Biosciences; UNC flow core) as previously described (Poulos et al., 2016; Xie et al., 2014). For sorting of LSK cells, total BM cells harvested from the femur, tibia and pelvis of 5 mice were combined and subject to c-KIT-APC+ selection (Miltenyi biotec). The obtained c-Kit positive selected cells are incubated with a lineage marker cocktail consisting of B220-FITC, CD11b-FITC, Gr1-FITC, CD3e-FITC, Ter119-FITC, CD8a-FITC, CD5-FITC, CD2-FITC and CD4-FITC (purchased from eBioscience as listed above), as well as the SCA-1-PE-Cy7 antibodies (BD Biosciences 558162), for 30 minutes. Sorting of LSK cells was carried out by the UNC flow core with a FACSAria II machine. For assaying apoptosis, the cells were incubated with Annexin-V FITC (Cell Signaling #6592) and analyzed as described before (Xie et al., 2014; Xu et al., 2015).

RNA-sequencing (RNA-seq) and data analysis. RNA-seq raw data were first trimmed to remove the Illumina adapters by using a custom script (UNC HTSF/Genomics core and UNC Cancer Center Bioinformatics core), and then mapped to the mouse reference genome using the HISAT2 with default parameters (Kim et al., 2015). Gene expression

levels were quantified with featureCounts (Liao et al., 2014) with default parameters that discarded multi-mapping reads. Raw gene counts were normalized, and differential expression calculated using DESeq2 with “betaPrior” turned on (Love et al., 2014). The MA plot of gene expression data was generated using the DESeq2 table and R.

Cells. HPC-7 cells, a cell line mimicking HSPCs (a kind gift of Prof. Leif Carlsson, Umeå University, Sweden), were cultured and maintained as previously described (Dahl et al., 2008; Wilson et al., 2010).

ChIP-sequencing (ChIP-seq). ChIP-seq was carried out as described before (Lu et al., 2016; Xu et al., 2015). The H3K4me3 antibody used for ChIP-seq was purchased from Abcam (ab8580).

ATAC-seq. ATAC-seq was carried out with the Epicentre Tn5 transposome and Illuminia kit according to the manufacturer protocol.

ATAC-seq data analysis. ATAC-seq reads were aligned to the mouse genome (mm9) using the software BWA (version “0.7.15-r1140”) and default parameters (Li and Durbin, 2010). The software MACS2 (Feng et al., 2012) was used for calling peaks from the individual replicates, with the “--nomodel --extsize 150 –q 0.1” option. The read counts within the called peaks were used for comparison across samples.

Gene Set Enrichment Analysis (GSEA), Gene Ontology (GO) analysis, and Ingenuity Pathway Analysis (IPA). DESeq2 normalized counts were used for GSEA by the javaGSEA program (Subramanian et al., 2005) with default parameters. Briefly, GSEA with the Molecular Signature Database as well as customized gene sets was carried out according to providers’ instructions as described before (Lu et al., 2016; Xu et al., 2015). GO was carried out as previously described (Lu et al., 2016; Xu et al., 2015). For IPA, the deregulated genes identified by RNA-seq were uploaded and

analyzed using the online IPA tool (www.qiagenbioinformatics.com) to determine the perturbed molecular networks.

RT-qPCR and ChIP-qPCR. RT-qPCR and ChIP-qPCR was carried out to examine the gene expression changes and genomic binding, respectively, as described before (Lu et al., 2016; Xu et al., 2015). Primer information was provided in supplemental table S2. BPTF antibodies (Bethyl #A300-973 and Millipore #ABE24) were used in ChIP assays.

Colony formation unit (CFU) assay. 300 of LSK cells freshly sorted from BM were mixed with the methylcellulose medium (StemCell Technologies; MethoCult GF M3434) and plated in triplicate as carried out before (Lu et al., 2016; Xu et al., 2015). For replating, approximately 5,000 cells isolated from cell colonies in the previous plating were seeded again in the same semi-solid medium. CFUs were counted every 10 days post-plating. Identity of each colony was defined by morphology according to manufacturer's specifications (StemCell Technologies).

shRNA and gene knockdown. The pLKO lentiviral constructs with the *Bptf* specific shRNA were purchased from Sigma (see below tables) and validated in our previous work (Zhou et al., 2016). HEK293 cells were used for producing virus as previously described (Lu et al., 2016; Xu et al., 2015).

sh_#1	TRCN0000238661	ACCGGATTATTACGGTGTTAT	mouse	CDS
sh_#2	TRCN0000238660	GACCGTAACCTCGTTCATATT	mouse	3UTR

Statistics. Data are presented as the mean ± SD of at least three independent experiments. Statistical analysis was performed with two-tailed Student's t test for comparing two sets of data with assumed normal distribution.

Reference of supplement document:

Bock, C., Beerman, I., Lien, W.H., Smith, Z.D., Gu, H., Boyle, P., Gnirke, A., Fuchs, E., Rossi, D.J., and Meissner, A. (2012). DNA methylation dynamics during in vivo differentiation of blood and skin stem cells. Molecular cell *47*, 633-647.
Dahl, L., Richter, K., Hagglund, A.C., and Carlsson, L. (2008). Lhx2 expression promotes self-renewal of a distinct multipotential hematopoietic progenitor cell in embryonic stem cell-derived embryoid bodies. PLoS ONE *3*, e2025.
Feng, J., Liu, T., Qin, B., Zhang, Y., and Liu, X.S. (2012). Identifying ChIP-seq enrichment using MACS. Nature protocols *7*, 1728-1740.
Kim, D., Langmead, B., and Salzberg, S.L. (2015). HISAT: a fast spliced aligner with low memory requirements. Nat Methods *12*, 357-360.
Li, H., and Durbin, R. (2010). Fast and accurate long-read alignment with Burrows-Wheeler transform. Bioinformatics *26*, 589-595.
Li, L., Jothi, R., Cui, K., Lee, J.Y., Cohen, T., Gorivodsky, M., Tzchori, I., Zhao, Y., Hayes, S.M., Bresnick, E.H., *et al.* (2011). Nuclear adaptor Ldb1 regulates a transcriptional program essential for the maintenance of hematopoietic stem cells. Nature immunology *12*, 129-136.
Liao, Y., Smyth, G.K., and Shi, W. (2014). featureCounts: an efficient general purpose program for assigning sequence reads to genomic features. Bioinformatics *30*, 923-930.
Love, M.I., Huber, W., and Anders, S. (2014). Moderated estimation of fold change and dispersion for RNA-seq data with DESeq2. Genome Biol *15*, 550.
Lu, R., Wang, P., Parton, T., Zhou, Y., Chrysovergis, K., Rockowitz, S., Chen, W.Y., Abdel-Wahab, O., Wade, P.A., Zheng, D., *et al.* (2016). Epigenetic Perturbations by Arg882-Mutated DNMT3A Potentiate Aberrant Stem Cell Gene-Expression Program and Acute Leukemia Development. Cancer cell *30*, 92-107.
Poulos, M.G., Ramalingam, P., Gutkin, M.C., Kleppe, M., Ginsberg, M., Crowley, M.J., Elemento, O., Levine, R.L., Rafii, S., Kitajewski, J., *et al.* (2016). Endothelial-specific inhibition of NF-kappaB enhances functional haematopoiesis. Nat Commun *7*, 13829.
Seita, J., Sahoo, D., Rossi, D.J., Bhattacharya, D., Serwold, T., Inlay, M.A., Ehrlich, L.I., Fathman, J.W., Dill, D.L., and Weissman, I.L. (2012). Gene Expression Commons: an open platform for absolute gene expression profiling. PLoS ONE *7*, e40321.
Subramanian, A., Tamayo, P., Mootha, V.K., Mukherjee, S., Ebert, B.L., Gillette, M.A., Paulovich, A., Pomeroy, S.L., Golub, T.R., Lander, E.S., *et al.* (2005). Gene set enrichment analysis: a knowledge-based approach for interpreting genome-wide expression profiles. Proceedings of the National Academy of Sciences of the United States of America *102*, 15545-15550.
Wilson, N.K., Foster, S.D., Wang, X., Knezevic, K., Schutte, J., Kaimakis, P., Chilarska, P.M., Kinston, S., Ouwehand, W.H., Dzierzak, E., *et al.* (2010). Combinatorial transcriptional control in blood stem/progenitor cells: genome-wide analysis of ten major transcriptional regulators. Cell stem cell *7*, 532-544.
Xie, H., Xu, J., Hsu, J.H., Nguyen, M., Fujiwara, Y., Peng, C., and Orkin, S.H. (2014). Polycomb repressive complex 2 regulates normal hematopoietic stem cell function in a developmental-stage-specific manner. Cell stem cell *14*, 68-80.
Xu, B., On, D.M., Ma, A., Parton, T., Konze, K.D., Pattenden, S.G., Allison, D.F., Cai, L., Rockowitz, S., Liu, S., *et al.* (2015). Selective inhibition of EZH2 and EZH1 enzymatic activity by a small molecule suppresses MLL-rearranged leukemia. Blood *125*, 346-357.
Zhou, Y., Wang, L., Vaseghi, H.R., Liu, Z., Lu, R., Alimohamadi, S., Yin, C., Fu, J.D., Wang, G.G., Liu, J., *et al.* (2016). Bmi1 Is a Key Epigenetic Barrier to Direct Cardiac Reprogramming. Cell stem cell *18*, 382-395.
